# Photoreversible stretching of a BAPTA chelator marshalling Ca^2+^-binding in aqueous media

**DOI:** 10.3762/bjoc.15.273

**Published:** 2019-11-21

**Authors:** Aurélien Ducrot, Arnaud Tron, Robin Bofinger, Ingrid Sanz Beguer, Jean-Luc Pozzo, Nathan D McClenaghan

**Affiliations:** 1 Institut des Sciences Moléculaires, CNRS UMR 5255, Univ. Bordeaux, 351 cours de la Libération, 33405 Talence, France

**Keywords:** azobenzene, BAPTA, calcium binding, photorelease, photoswitch

## Abstract

Free calcium ion concentration is known to govern numerous biological processes and indeed calcium acts as an important biological secondary messenger for muscle contraction, neurotransmitter release, ion-channel gating, and exocytosis. As such, the development of molecules with the ability to instantaneously increase or diminish free calcium concentrations potentially allows greater control over certain biological functions. In order to permit remote regulation of Ca^2+^, a selective BAPTA-type synthetic receptor / host was integrated with a photoswitchable azobenzene motif, which upon photoirradiation would enhance (or diminish) the capacity to bind calcium upon acting on the conformation of the adjacent binding site, rendering it a stronger or weaker binder. Photoswitching was studied in pseudo-physiological conditions (pH 7.2, [KCl] = 100 mM) and dissociation constants for azobenzene *cis*- and *trans*-isomers have been determined (0.230 μM and 0.102 μM, respectively). Reversible photoliberation/uptake leading to a variation of free calcium concentration in solution was detected using a fluorescent Ca^2+^ chemosensor.

## Introduction

In terms of synthetic calcium binding molecules, since the 1,2-bis(*o*-aminophenoxy)ethane-*N*,*N*,*N*',*N*'-tetraacetic acid (BAPTA) scaffold was first described by Tsien, it has been widely used in biological systems and has given rise to a wealth of derivatives [1]. Key to the wide interest in this cation binder are its high specificity for Ca^2+^ ions over Mg^2+^ ions, and relative insensitivity to pH in biological media due to the relatively low p*K*_a_ of the electron-poor aniline nitrogens [1]. BAPTA-type molecules complex Ca^2+^ in an octacoordinated fashion, involving the two aniline functions, the two central ether oxygens and the four carboxylates, as elucidated by the crystallographic structure of a 1:1 host–guest BAPTA system [2]. This moiety has been exploited in the development of various fluorescent supramolecular chemosensor systems and even molecular logic systems [3–11].

Equally, the incorporation of photochemically active groups has been used to trigger the decrease of the ligand’s affinity for calcium ions leading to photorelease [12–14], and as such has proved an alternative to C–N-bond photocleavage [15–16]. A molecular prototype for the photodecaging of calcium was Nitr-5, where an electron-withdrawing carbonyl function is generated from a secondary alcohol adjacent to the BAPTA binding site upon photoirradiation, which proved sufficient to lower the binding affinity 40-fold and liberate calcium in biological media [17]. This design was subsequently improved in a symmetrical variant and indeed a wealth of successful variants for photodecaging calcium have been described including examples whose photochemical quantum yield approaches unity [18–24].

While photorelease of calcium can be efficiently achieved in micro-to-millimolar concentration and has led to new insights in neurobiology, reversible uptake and release could give rise to calcium fluxes which would give greater control over cellular function or ultimately provide new information on the role of calcium. With this long-term objective in mind, photochromic groups have been integrated with BAPTA (or structurally related EDTA) such that photoswitching would enhance or lower Ca^2+^ binding based on the state of the adjacent photoswitch. Photogeneration of a positive charge proximal to the BAPTA site is undoubtedly the most robust approach to provoke calcium cation release, while diminishing electron density on the chelating groups would also have a significant effect. Some groups reported BAPTA-diarylethene and EDTA-spiropyran conjugates, while noting limitations for implementation in terms of fatigue, solubility and relatively small affinity changes [25–26]. We further highlight a structurally elegant design integrating a spiroamidorhodamine with a BAPTA, whose anticipated switching remains to be proven [27]. Further, interesting approaches are currently being developed on interfacing calmodulin, a messenger protein ionophore, with photochromes [28].

**Figure 1:**
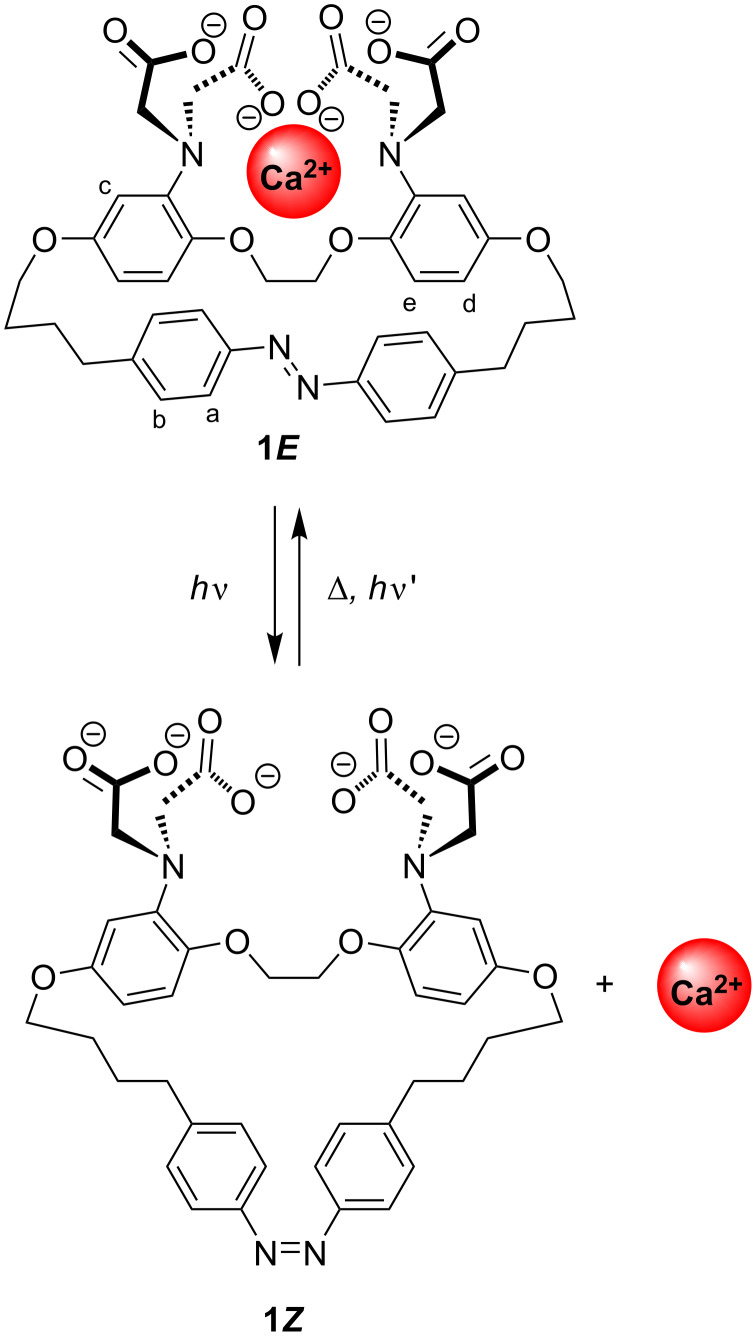
Azobenzene-BAPTA **1*E*** and **1*Z*** (a, b, c, d and e denote specific protons), showing idealized Ca^2+^ uptake and release. Counter ions omitted for clarity.

The use of steric effects to decrease binding offers an alternative approach to generate calcium release, where deforming an ion-binding site would render it less well-adapted to bind a guest. Photoisomerization of the azobenzene moiety has previously been successfully used to modulate the affinity of crown ethers [29], lariats [30], and foldamers [31] for various ions and switch between complexes of different molecularity, albeit largely in organic solvents [32]. Azobenzene is generally considered relatively resistant to fatigue and has been employed to evoke changes in different biological systems, including ion channels based on changes of properties between *cis* and *trans*-forms [33–34]. Herein we describe a BAPTA host molecule **1**, with an azobenzene moiety integrated in the tether linking both aromatic rings, as illustrated in Figure 1. The principle goal of the current work, with a long-term view of interfacing biological systems, was the development of a water-soluble molecule which has a high affinity and selectivity for calcium, allowing binding of physiological levels of calcium, whose binding can be switched reversibly as efficiently as possible both in terms of affinity and quantum yield. In principle this would offer real-time regulation of free calcium levels. In the current design, the elongated **1*E*** form of azobenzene in the synthetic BAPTA podand can adopt the preferred binding geometry to sequester Ca^2+^, while the metastable **1*Z*** is anticipated to deform the chelating site separating the binding groups, leading to a lowered guest affinity. Herein we describe the synthesis of **1**, its photoswitching, and affinity changes between both forms in pseudo-physiological conditions, further rationalized by energy-minimized molecular modelling structures.

## Results and Discussion

**Scheme 1:**
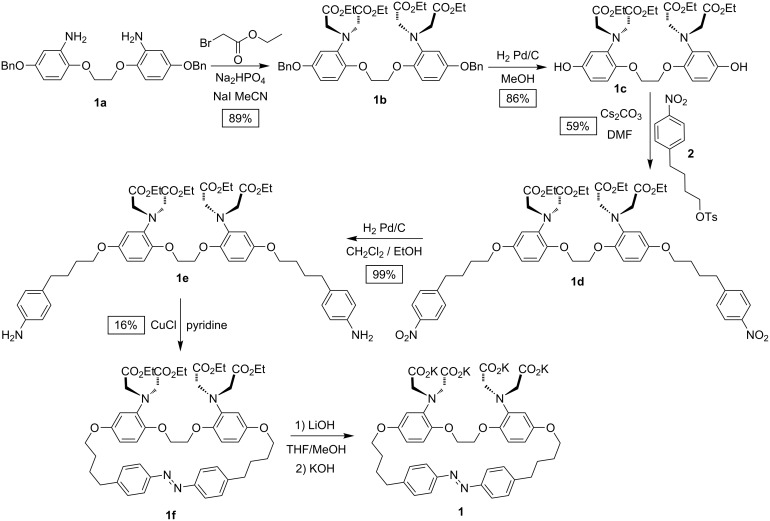
Synthesis of azobenzene-tethered BAPTA **1**.

The synthetic pathway for the preparation of molecule **1** is shown in Scheme 1 and detailed synthetic procedures are given below. Briefly, the synthesis of **1** started with the preparation of a BAPTA core via a multistep route adapting the synthetic route developed by Crossley et al. [35]. 1,4-Hydroquinone was benzylated and subsequently nitrated. This intermediate then underwent a regioselective monodeprotection to generate a lone phenol group. The phenolate was reacted with 1,2-dibromoethane and the nitrobenzene groups were reduced to the corresponding anilines giving **1a**. The double aniline **1a** was alkylated using ethyl bromoacetate under basic conditions in acetonitrile, forming the BAPTA precursor **1b**. The palladium-catalyzed benzyl cleavage was followed by a double alkylation with **2**, reactant **2** being synthesized from 4-(4-nitrophenyl)butanoic acid. The 4-nitrobenzene groups of the pendant arms were subsequently reduced yielding the corresponding anilines (**1e**). A cyclization reaction yielding the corresponding azobenzene **1f** was performed using a Cu(I) catalyst generated in situ [36]. Finally, the esters were hydrolyzed under mild conditions resulting in the azobenzene-BAPTA macrocycle **1**.

**Figure 2:**
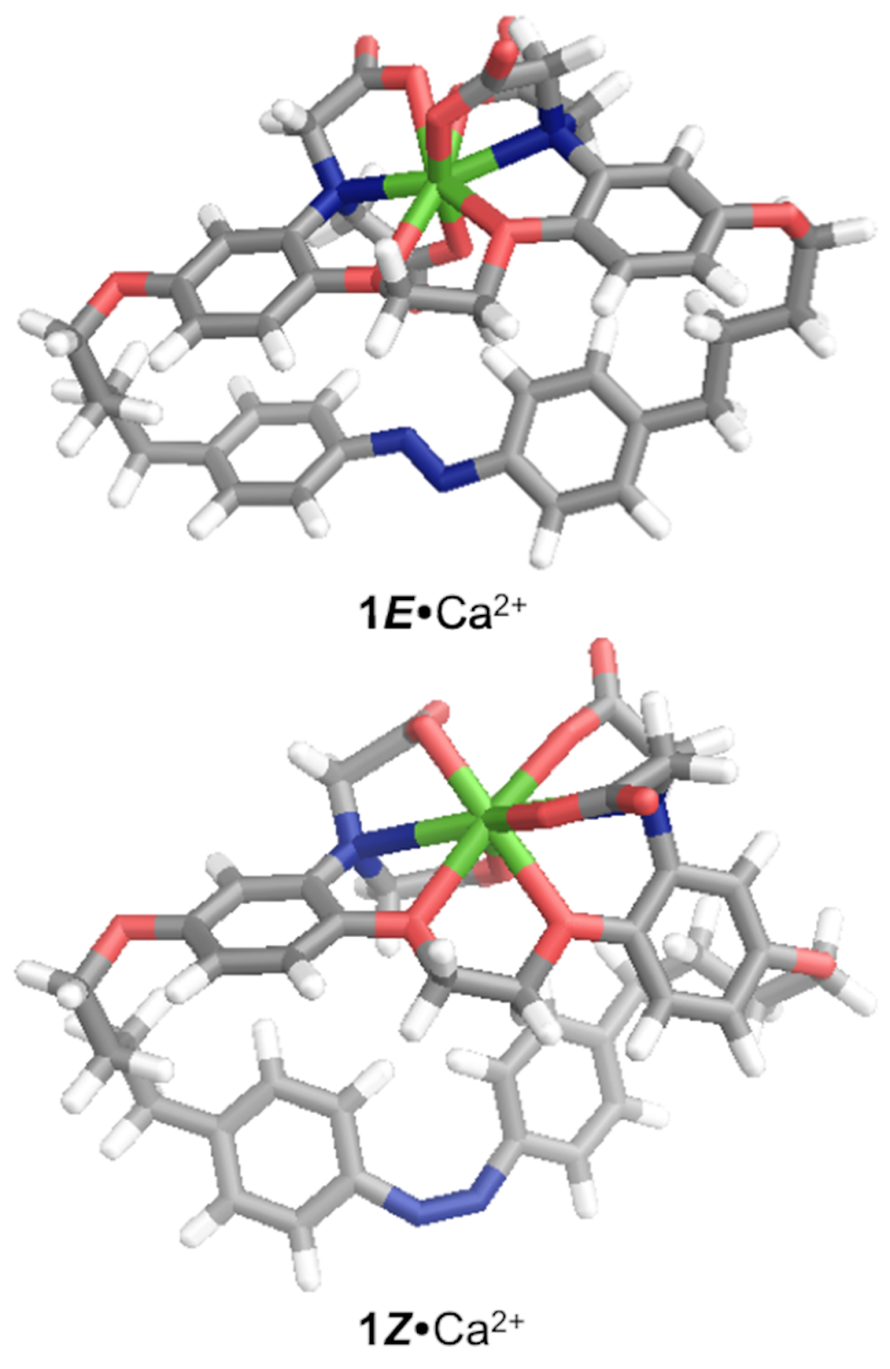
Energy-minimized molecular modelling structures of **1*E•***Ca^2+^ and **1*Z•***Ca^2+^ (PM6).

To gain some insight into the possible structures of the **1*E*** and **1*Z*** chelators and differences between them, which may have consequences on the chelation, energy-minimized (PM6) structures were determined (Figure 2). The *trans-*form was found to be the lowest energy structure, as is generally observed for azobenzenes [37]. In the *trans-* versus *cis-*chelates the following bond lengths were noted: N–Ca^2+^: 2.35 and 2.49 Å cf. 3.24 and 3.03 Å. Smaller differences were noted considering the COO^−^–Ca^2+^: 2.20, 2.19, 2.29 and 2.70 Å cf. 2.21, 2.21, 2.23 and 2.23 Å or the ether linkage O–Ca^2+^: 2.44 and 2.49 Å cf. 2.46 and 2.51 Å. Most significantly, the aniline N–N distance is stretched from 4.71 Å for the *trans-*form to 5.96 Å for the *cis-*form, which, taken in conjunction with the lengthened N–Ca^2+^ bond lengths tends to suggest a less tight calcium binding in the photogenerated *cis*-form as compared to the *trans*-form, consistent with photo-promoted guest release.

**Figure 3:**
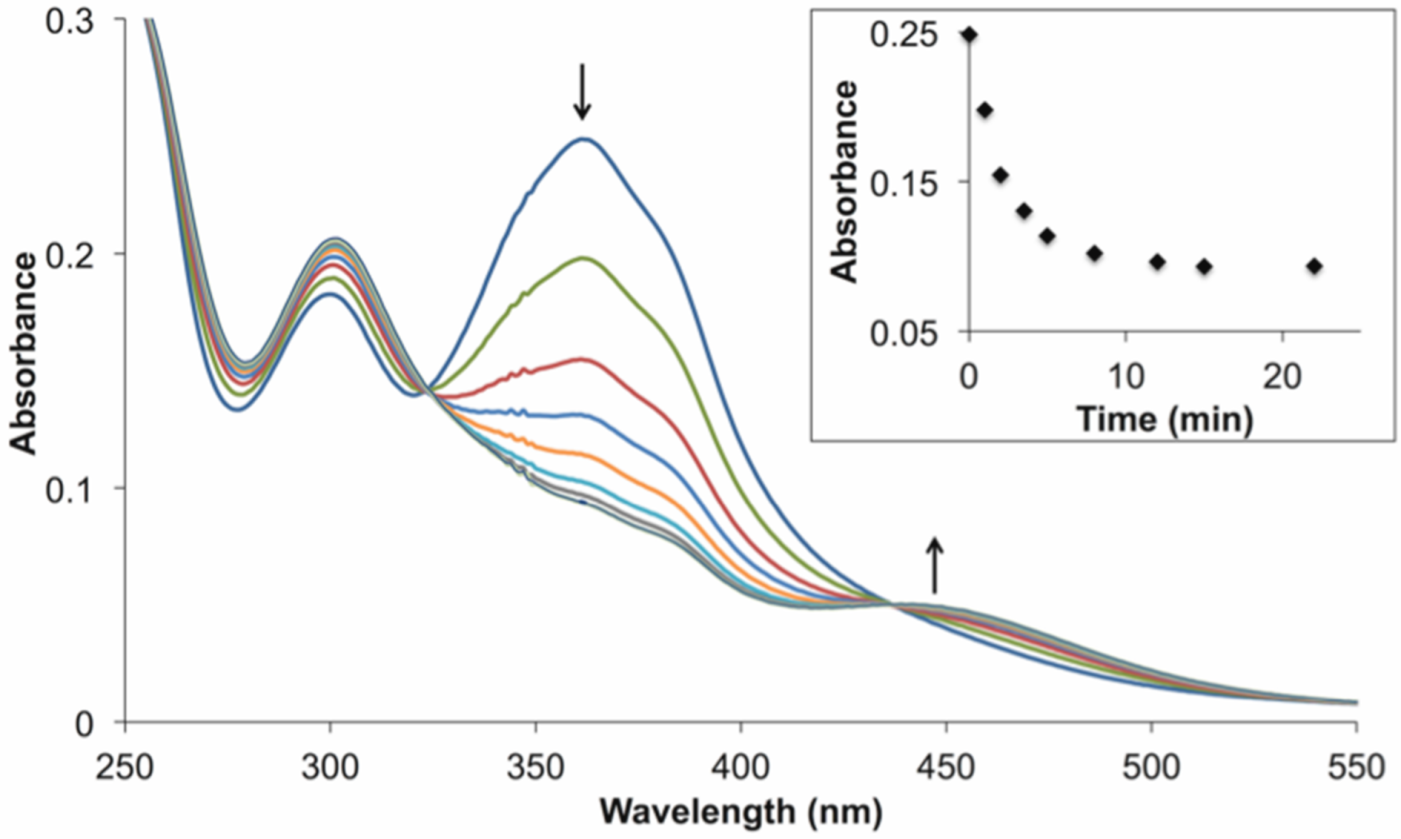
Electronic absorption spectra showing changes associated with photoisomerization of **1*E*** (40 μM) to **1*Z*** in aqueous 0.03 M MOPS buffer at pH 7.2 in the presence of KCl (0.1 M) and EGTA (0.011 M). Inset: Absorbance at 362 nm as a function of irradiation time.

**Figure 4:**
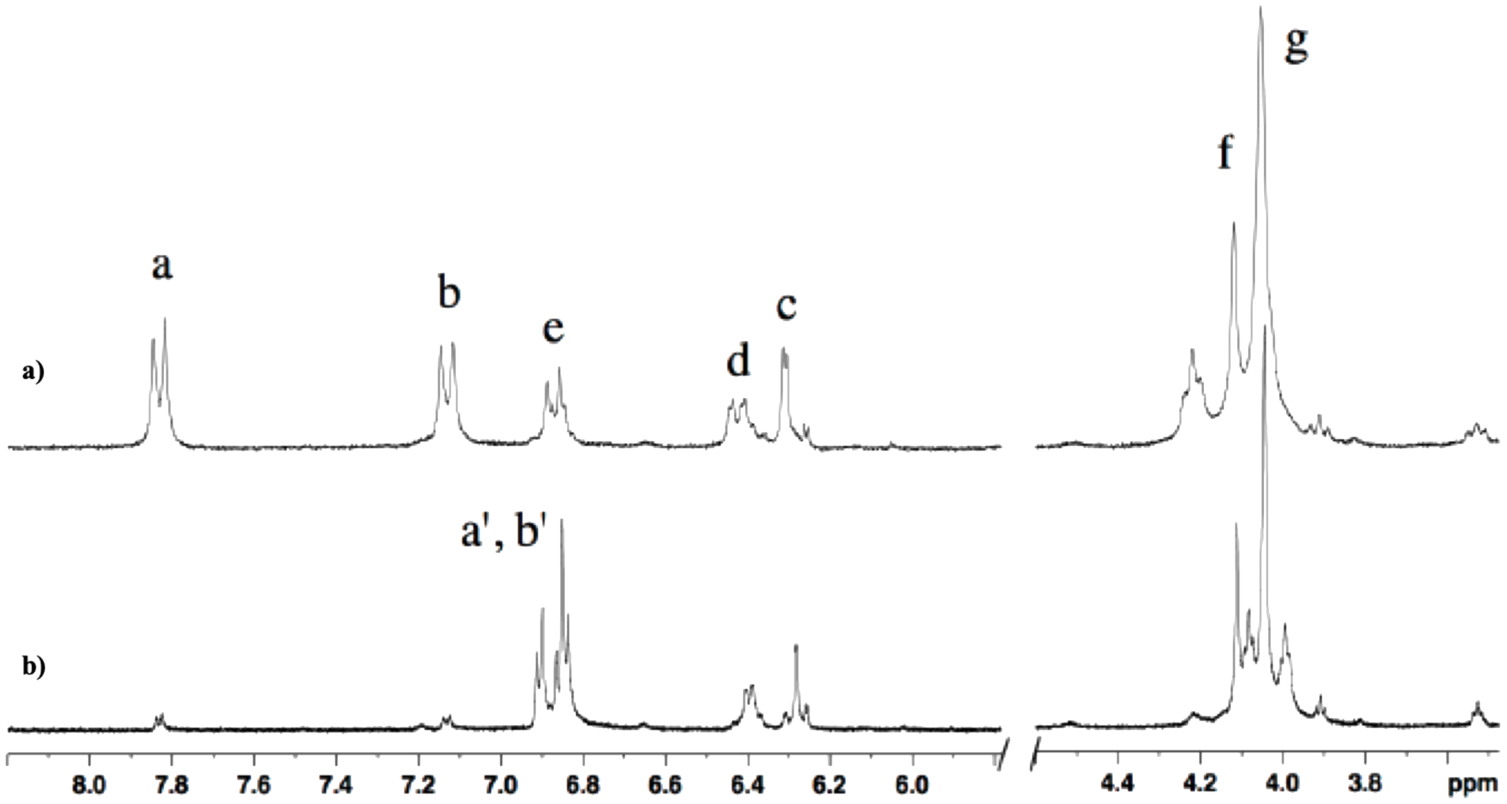
^1^H NMR spectra (300 MHz) recorded at room temperature (298 K) in D_2_O of a) the thermodynamically stable *trans-***1*E*** and b) at the photostationary state (PSS) after irradiation at 365 nm (88% of **1*Z*** and 12% of **1*E*** at PSS).

Exclusively *E*-isomers are observed in the ^1^H NMR spectra of the azobenzene-containing macrocycles **1f** and **1** at 25 °C (Supporting Information File 1, Figures S13 and S14). Next, in the absence of Ca^2+^ ions, **1*E*** was subjected to irradiation into the π–π* band (λ_max_ = 362 nm) and photoisomerization of **1*E*** to **1*Z*** took place, as evidenced by the decrease of the π–π* absorption in the range 330-440 nm and the increase of the *n*–π* absorption above 440 nm (Figure 3), equally observed for **1f*E*** to **1f*Z*** (Figure S1 in Supporting Information File 1). The NMR analysis of photoirradiated samples of macrocyles **1** showed that the photostationary state (PSS) for the E→Z photoisomerization at 365 nm was 88:12 in favor of the *cis-*form (Figure 4).

**Figure 5:**
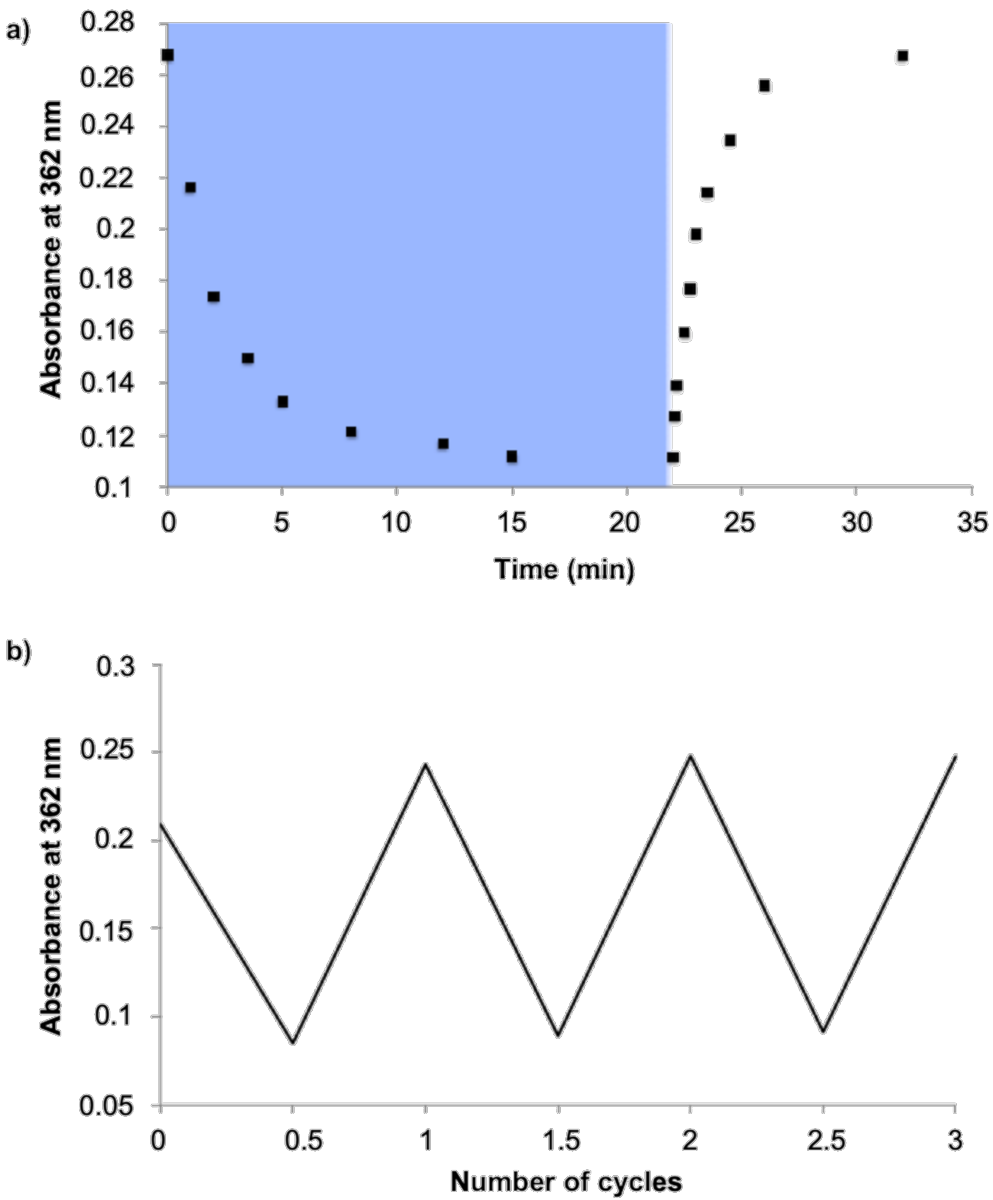
a) Multiple *trans–cis* cycles of **1*E*** (40 μM) indicated by absorption changes at 362 nm in aqueous 0.03 M MOPS buffer at pH 7.2 in presence of KCl (0.1 M) and EGTA (0.011 M). Each cycle corresponds to irradiation at 365 nm (blue), followed by thermal return at room temperature (white). b) Fatigue study of **1*E*** (40 μM) after 3 cycles.

The reversibility of the photoswitching of the supramolecular host was tested over several isomerization cycles with little evidence of fatigue (Figure 5). Thermal isomerization *Z*→*E* resulted in the restoration of the electronic absorption band attributed to the π–π* transition at 30 °C (Figure 5), the rate constant (*k*) was estimated at 5.4 × 10^−5^ s^−1^. Photoisomerization was also recorded in electron-absorption spectroscopy in the presence of calcium (see Figure S2 in Supporting Information File 1). The presence of the ion has the effect of reducing the reversibility of the isomerization cycles due to an apparent accelerated degradation, which may be attributed to rendering an unwanted photochemical pathway more competitive. The fatigue study showed that more than 90% of **1*E*** were recovered after each cycle (Figure S3, Supporting Information File 1). On repeating this cycle, slow decomposition was observed, estimated at 14% after 3 cycles.

**Figure 6:**
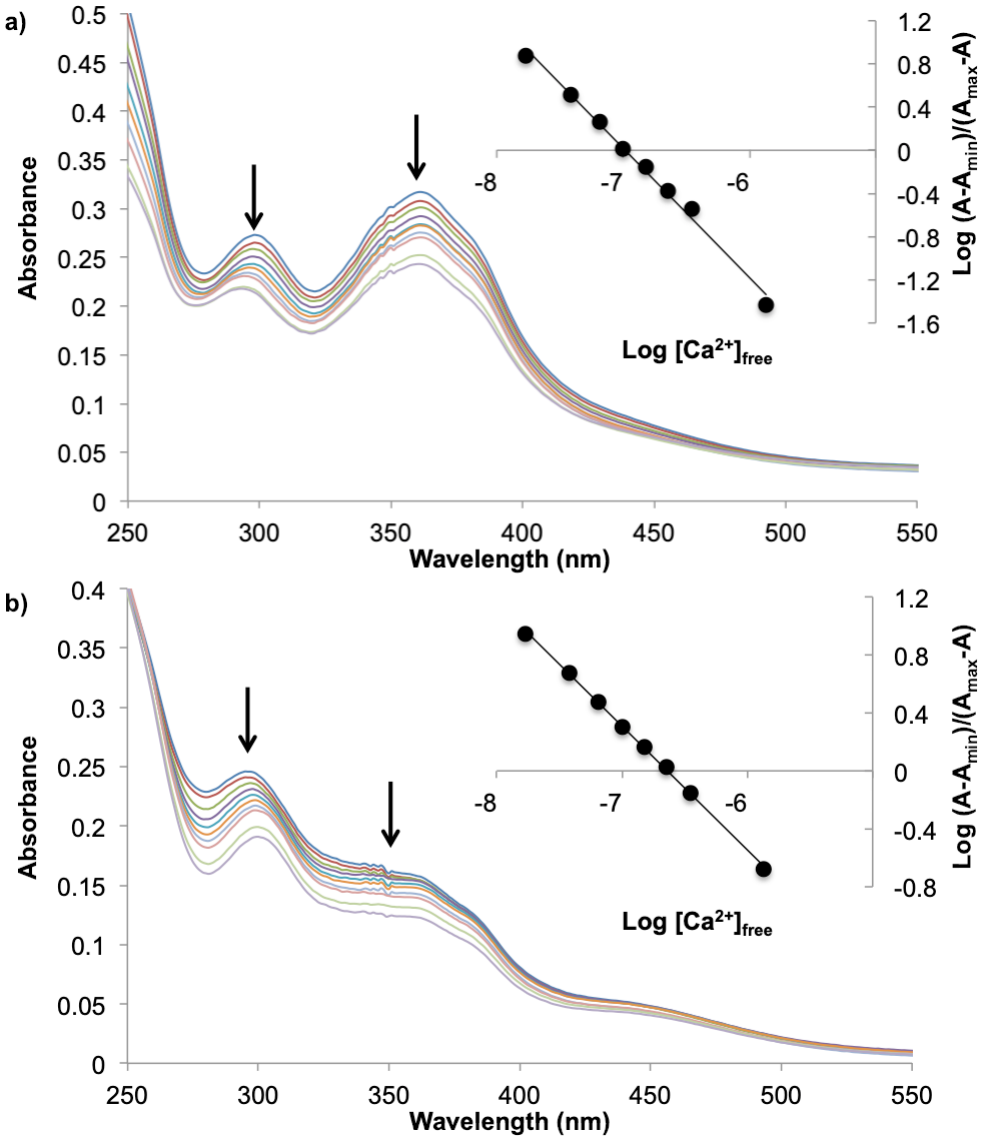
Electronic absorption spectra changes of **1*E*** (42 μM) (a) and **1*Z*** (43 μM) (b) in aqueous 0.03 M MOPS buffer at pH 7.2 in presence of KCl (0.1 M), and EGTA (0.010 M) upon Ca^2+^ addition (free Ca^2+^ concentration = 0.017, 0.038, 0.065, 0.100, 0.150, 0.225, 0.35, 1.35 and 39 μM). Insets: Hill plots for the absorbance change at 305 nm as a function of free Ca^2+^, affording log *K*_d_ (*x*-axis intercept).

Calcium binding by hosts **1*E*** and **1*Z*** was investigated by spectrophotometry, specifically monitoring aromatic ring chromophores which give rise to absorption bands observed in the UV, assigned to *n*–π* and π–π* transitions [38]. The calcium complexation studies of **1*E*** and **1*Z*** were performed under pseudo-intracellular conditions (100 mM KCl, 30 mM MOPS, pH 7.2). In the absence of ions, the spectra of **1*E*** (Figure 6a) comprised absorption bands at 298 nm (ε: 5815 M^−1^ cm^−1^) and 359 nm (ε: 6728 M^−1^ cm^−1^). The complexation of Ca^2+^ (Figure 6a) induced a blue-shifting and a decrease of the absorption bands of **1*E*** at 291 nm (ε: 4748 M^−1^ cm^−1^) and 355 nm (ε: 5230 M^−1^ cm^−1^). The spectrum of **1*Z*** (Figure 6b) exhibited two absorption bands at 303 nm (ε: 5450 M^−1^ cm^−1^) and 348 nm (ε: 2700 M^−1^ cm^−1^). The complexation of Ca^2+^ (Figure 6b) induced blue-shifting and a decrease of the absorption band intensities of **1*E*** at 297 nm (ε: 4900 M^−1^ cm^−1^) and 345 nm (ε: 3000 M^−1^ cm^−1^). After linearizing the spectral changes via a Hill plot, a 1:1 binding with a *K*_d_ of 0.102 μM in the case of **1*E*** and 0.230 μM for **1*Z*** were determined. The value for **1*E*** is similar to the *K*_d_ value reported for BAPTA (*K*_d_ = 0.110 μM) and importantly calcium binding by the *cis-*form was circa 2.5-fold less than that of the *trans-*form [39]. This is conducive to photopromoted ion release.

Chemical actinometry afforded the quantum yield of the photoisomerization reaction (λ_ex_ = 365 nm) [40]. For solutions of **1*E*** in the absence and presence of Ca^2+^, the measured quantum yields were identical (0.08) suggesting that the ion does not influence the efficiency of the photoreaction.

**Figure 7:**
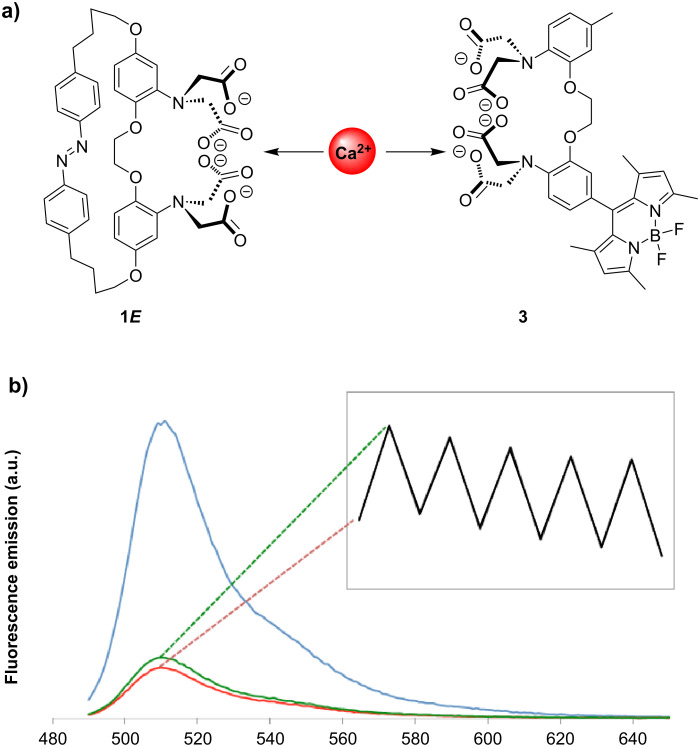
a) Reversible Ca^2+^ exchange between photoregulated host **1** and turn-“on” fluorescent probe **3**. b) Blue: **3** (5 µM) in 0.1 M KCl and added Ca^2+^ (5 µM) in aqueous 0.03 M MOPS buffer at pH 7.2, red: after the addition of 50 µM **1*E***, green: after photoirradiation at 365 nm. Inset: fluorescent response over 5 cycles.

In order to unambiguously prove the liberation of Ca^2+^ upon photexcitation in solution, azobenzene macrocyle **1** was used as a reversible Ca^2+^ photoejector in the presence of a Ca^2+^-selective “turn-on” fluorescent probe (**3**, Figure 7) [41]. The fluorescence of BODIPY dye **3** in the absence of calcium, is quenched by an intramolecular photoinduced electron-transfer reaction, while ion binding blocks this quenching pathway restoring emission. Thus, ion liberation from **1** and transfer to **3** would result in a fluorescence off–on switching of the latter.

To perform the ion-transfer experiment, the fluorescent dye is loaded with one equivalent of Ca^2+^, giving rise to a highly fluorescent species upon excitation at 485 nm. The fluorescence is greatly diminished after the addition of 10 equivalents of **1*E***, as the azobenzene macrocycle abstracts the Ca^2+^ ion from fluorophore **3**. Upon irradiation of the ensemble at 365 nm at these low concentrations, a small but reproducible increase in fluorescence intensity can be observed, directly corresponding to the lower complexation/release of the Ca^2+^ ions in the PSS (Figure 7b). Furthermore, several irradiation cycles were successfully completed with this reversible system. The fatigue study showed that more than 90% of the fluorescence enhancement was recovered after each cycle despite a slight decrease of fluorescence intensity due to some photoinstability estimated at 20% after 5 cycles (inset Figure 7b).

The aforementioned experiment is simply a demonstration that calcium can be reversibly taken up and released in solution, dilute solutions being convenient to simultaneously monitor the state of the azobenzene and the fluorescence. In terms of possible applications for reversible photodecaging in biological systems, the amount of calcium required to be released to evoke a change is very system dependent and as such it is impossible to generalize precise quantities (although they would invariably involve higher concentrations). However, we can take skeletal muscle fibers as a representative example, where an increased free calcium concentration promotes muscular contraction [16]. It is noted that 5 μM of free calcium is in principle sufficient to induce a contraction (resting concentration of Ca^2+^ being 0.19–0.28 μM), but that the effective concentration should be much higher as the fibers themselves can buffer free calcium. This was effectively achieved (non-reversibly) with mM concentrations of the calcium-cage nitrophenyl-EGTA (*K*_d_ of 0.080 μM on excitation at 350 nm with a quantum yield of 0.23) [16]. For comparison, molecule **1** has a similar *K*_d_ and the quantum yield is in the same order of magnitude as nitrophenyl-EGTA but is around 3 times lower, thus in principle it could sequester calcium sufficiently well and show sufficient photoactivity in this situation. However, the modest 2.5-fold difference of binding affinity between complexing and non-complexing forms, while comparable to the best-known photochromic variants [25–26], would need to be significantly increased (at least by an order of magnitude) to have sufficiently efficient ion release and free calcium increase for biological application. Indeed, this is the major challenge for photoswitchable variants, efficient release being more dramatic and readily achieved in versions with photodegradable, and hence non-switchable binding sites.

## Conclusion

In conclusion, a photochromic BAPTA-based calcium ion host (**1**) incorporating an azobenzene photochrome is described. This chelating molecule showed high affinity for Ca^2+^ under pseudo-physiological conditions (*K*_d_ = 0.102 μM) and showed efficient photochemical *trans-*to-*cis* switching in terms of quantum yields (0.08) and PSS composition (88% *cis*). The calcium binding affinity of the *cis*-form was diminished by circa 2.5-fold with respect to the *trans-*form as the chelator was stretched rendering the binding pocket ill-adapted to accommodate the calcium, as suggested by molecular modelling. Reversible Ca^2+^ liberation and subsequent uptake was evidenced using a fluoroionophore. While the simultaneous observation of fluorescence and absorption was conveniently followed at low concentration, any future biological practical use would undoubtedly require use at much higher concentrations. In this regard a higher release efficiency would equally undoubtedly be required than that shown by prototype **1** described herein. To this end, shortening/rigidification of the hydrocarbon azobenzene-BAPTA linkages in **1** may be anticipated to lead to an exalted difference in *trans*/*cis-*binding affinity. However, in our hands, the synthesis of such architectures using protocols similar to those described herein proved inefficient in these more sterically challenging variants.

## Experimental

### Materials and methods

All synthetic steps were performed under a dry nitrogen atmosphere using standard techniques. Commercially available reagents and solvents were used as received unless otherwise stated. THF and diethyl ether were distilled over sodium/benzophenone. Acetonitrile and dichloromethane were distilled over calcium hydride immediately before use. Compounds **1a** and **2** were synthesized according to literature procedures [35,42]. The progress of all reactions was monitored by thin layer chromatography on silica gel 40 F254. Column chromatography was performed on silica gel 40 (0.230–0.400 mm or 40–63 μm,). ^1^H and ^13^C NMR experiments were performed at 295 K on the following spectrometers: Bruker DPX 200 (^1^H: 200 MHz) or an Avance 300 (^1^H: 300 MHz, ^13^C: 75 MHz) spectrometer. Chemical shifts are reported in ppm (δ) and are referenced to the NMR solvent residual peaks. Abbreviations used are s = singlet, d = doublet, t = triplet, q = quartet, m = multiplet. Reagent grade tetrahydrofuran (THF) was distilled under argon over sodium benzophenone ketyl. CH_2_Cl_2_ was distilled over CaH_2_ under argon. Mass spectrometry was performed at the CESAMO analytical center (University of Bordeaux, France) on a QStar Elite mass spectrometer (Applied Biosystems). The instrument is equipped with an electrospray ion (ESI) source and spectra were recorded in the positive mode. High-resolution mass spectrometry (HRMS) measurements were performed with an ESI source on a Q-TOF mass spectrometer with an accuracy tolerance of 2 ppm (Fédération de Recherche CBM/ICOA (FR2708) platform). The electrospray needle was maintained at 5000 V and operated at room temperature. Samples were introduced by injection through a 20 μL sample loop into a 4500 μL/min flow of methanol from the LC pump. Electronic absorption spectra were measured on a Varian Cary 5000 UV–vis–NIR spectrophotometer. Steady-state emission spectra were recorded on a spectrofluorometer fitted with a PMT detector and exciting with a 450 W Xe lamp across a double monochromator, and were corrected for instrumental response. Photoreaction quantum yields were determined upon excitation at 365 nm using the couple potassium ferrioxalate–phenanthroline as a chemical actinometer on an optical bench equipped with a 150 W Hg–Xe lamp and a monochromator [40]. Samples (40 μM) were stirred during the irradiation and the amount of converted material was determined at 2 min intervals by UV–vis spectroscopy following the disappearance of the band at 362 nm. The error in the photoreaction quantum yield determination was estimated at ±15%. Calcium titration: Two calcium calibration stock solutions were prepared, one containing 10 mM EGTA (0 μM Ca^2+^ buffer) and another with 10 mM CaEGTA (39 μM Ca^2+^ buffer). In addition, both buffers were charged with 100 mM KCl and 30 mM 3-(*N*-morpholino)-propanesulfonic acid (MOPS) adjusted at pH 7.2. In both buffers an equivalent amount of the Ca^2+^-binding probe was dissolved with a final concentration between 1–10 μM. In order to determine the dissociation constant (*K*_d_), spectra have been recorded after having adjusted the free Ca^2+^ concentration to 0.017 μM, 0.038 μM, 0.065 μM, 0.100 μM, 0.150 μM, 0.225 μM, 0.35 μM, 1.35 μM and 39 μM. Therefore, 2 mL of the 0 μM Ca^2+^ sample was placed in a cuvette and the spectra were recorded. Then, 200 μL of the sample were discarded and replaced with 200 μL of the 39 μM Ca^2+^ sample. In that fashion a concentration of 0.017 μM Ca^2+^ can be obtained without changing the concentration of the analyte. The aforementioned Ca^2+^ concentrations were then adjusted by discarding 250, 222, 250, 286, 333, 400, 500, 667 and 1000 μL and replacing the removed volume with an equivalent volume of the 39 μM Ca^2+^ sample. For the calculation of the free Ca^2+^ concentration a constant room temperature of 21 °C was assumed. The spectral change at a given wavelength is then introduced into Hill plot, where log (A − A_min_)/(A_max_ − A) is plotted against the logarithm of the free Ca^2+^ concentration as obtained from the Ca^2+^ buffers and A is the absorption at a certain wavelength. The *x*-intercept gives the log *K*_d_. Molecular modelling: The molecular structure of **1*E***·Ca^2+^ and **1Z**·Ca^2+^ were built with AMPAC 10.1. A geometry optimization was performed by energy minimization using the PM6 method; solvation was not considered.

### Synthetic procedures

**Tetraethyl 2,2',2'',2'''-(1,2-ethanediylbis{oxy[5-(benzyloxy)-2,1-phenylene]nitrilo})tetraacetate (1b).** A mixture of 2,2'-[1,2-ethanediylbis(oxy)]bis[5-(benzyloxy)aniline] [20] (**1a**, 3 g, 6.57 mmol), ethyl bromoacetate (9 mL, 78.87 mmol), Na_2_HPO_4_ (4.72 g, 33.25 mmol) and NaI (0.66 g, 4.37 mmol) in 90 mL acetonitrile was heated under reflux conditions and the reaction was followed by TLC (SiO_2_, pentane/AcOEt 7:3, v/v). After 48 h, the reaction was allowed to cool to room temperature and the solvent was removed in vacuo. To the residue, 200 mL water was added and the aqueous solution extracted with toluene (3 × 100 mL). A final crystallization from EtOH yielded the title compound (4.69 g, 89%). ^1^H NMR (CDCl_3_, 200 MHz) δ 7.30–7.42 (m, 10H, C*H_Ar_*), 6.75 (d, *J* = 9.2 Hz, 2H, C*H_Ar_*), 6.43–6.48 (m, 4H, C*H_Ar_*), 4.97 (s, 4H, C*H_2_*), 4.18 (s, 4H, C*H_2_*), 4.14 (s, 8H, C*H_2_*), 4.06 (q, *J* = 7.2 Hz, 8H, C*H_2_*), 1.16 (t, *J* = 7.2 Hz, 12H, C*H_3_*) ppm; ^13^C NMR (CDCl_3_, 75 MHz) δ 170.5, 152.8, 143.9, 139.7, 136.4, 127.6, 126.9, 126.6, 113.8, 106.4, 105.6, 69.6, 67.1, 59.9, 52.6, 13.2 ppm; HRMS–ESI (*m*/*z*): [M + Na]^+^ calculated for C_44_H_52_N_2_O_12_Na, 823.3429; found, 823.3412.

**Tetraethyl 2,2',2'',2'''-{1,2-ethanediylbis[oxy(5-hydroxy-2,1- phenylene)nitrilo]tetraacetate (1c)**. A solution of **1b** (4.13 g, 5.16 mmol) in methanol (150 mL) and 10% Pd/C (0.5 g) was stirred vigorously under a H_2_ atmosphere for 24 h at room temperature. The reaction mixture was subsequently filtered over celite and the celite pad was washed with hot EtOAc. Column chromatography (SiO_2_, EtOAc/petroleum ether 7:3, v/v) yielded the title compound (2.76 g, 86%). ^1^H NMR (CDCl_3_, 300 MHz) δ 6.51 (d, *J* = 8.6 Hz, 2H, C*H_Ar_*), 6.33 (d, *J* = 2.7 Hz, 2H, C*H_Ar_*), 6.24 (dd, *J* = 8.6 Hz, *J* = 2.7 Hz, 2H, C*H_Ar_*), 5.86 (br. s, 2H, O*H*), 4.04–4.16 (m, 20H, C*H_2_*), 1.17 ( t, *J* = 7.2 Hz, 12H, C*H_3_*) ppm; ^13^C NMR (CDCl_3_, 75 MHz) δ 171.9, 150.9, 144.0, 140.4, 115.7, 108.0, 106.7, 68.0, 61.1, 53.7, 14.2 ppm; HRMS–ESI (*m*/*z*): [M + Na]^+^ calcd for C_30_H_40_N_2_O_12_Na, 643.2468; found, 643.2573.

**Tetraethyl 2,2',2'',2'''-[1,2-ethanediylbis(oxy{5-[4-(4-nitrophenyl)butoxy]-2,1-phenylene}nitrilo)]tetra acetate (1d)**. A solution of **1c** (0.60 g, 0.97 mmol), Cs_2_CO_3_ (1.26 g, 3.87 mmol) and **2** (1.35 g, 3.87 mmol) in 10 mL DMF was heated overnight at 110 °C. After allowing the solution to cool to room temperature, the solvent was removed in vacuo and the residue redissolved in DCM. The organic layer was washed with 20 mL H_2_O and subsequently dried over MgSO_4_. Column chromatography (SiO_2_, 1: DCM/EtOAc 95:5 → 85:15, v/v; 2: petroleum ether/EtOAc 1:1, v/v) yielded the title compound (0.55 g, 59%). ^1^H NMR (CDCl_3_, 300 MHz) δ 8.15 (d, *J* = 8.9 Hz, 4H, C*H_Ar_*), 7.37 (d, *J* = 8.9 Hz, 4H, C*H_Ar_*), 6.79 (d, *J* = 8.5 Hz, 2H, C*H_Ar_*), 6.38–6.46 (m, 4H, C*H_Ar_*), 4.20 (s, 4H, C*H_2_*), 4.18 (s, 8H, C*H_2_*), 4.10 (q, *J* = 7.1 Hz, 8H, C*H_2_*), 3.88–3.94 (m, 4H, O-C*H_2_*), 2.76–2.83 (m, 4H, C*H_2_*), 1.74–1.88 (m, 8H, C*H_2_*), 1.18 (t, *J* = 7.1 Hz, 12H, C*H_3_*) ppm; ^13^C NMR (CDCl_3_, 75 MHz) δ 171.4, 153.9, 150.2, 146.4, 144.7, 140.6, 129.2, 123.6, 123.2, 115.0, 107.1, 106.1, 68.1, 67.8, 60.7, 53.5, 35.5, 28.9, 27.5, 14.1 ppm; HRMS–FD (*m*/*z*): [M]^+^ calcd for C_50_H_62_N_4_O_16_ 974.4161; found, 974.4144.

**Tetraethyl 2,2',2'',2'''-[1,2-ethanediylbis(oxy{5-[4-(4-aminophenyl)butoxy]-2,1-phenylene}nitrilo)]tetraacetate (1e).** A mixture of **1d** (0.18 g, 0.23 mmol), 10% Pd/C (0.02 g) and a few drops of triethylamine in 100 mL DCM/EtOH 1:1 (v/v) was stirred vigorously under a hydrogen atmosphere for 48 h. The reaction mixture was filtered over celite and the celite pad washed with hot EtOAc. Column chromatography (SiO_2_, EtOAc) yielded the title compound (0.17 g, 99%). ^1^H NMR (CDCl_3_, 300 MHz) δ 7.02 (d, *J* = 8.2 Hz, 4H, C*H_Ar_*), 6.80 (d, *J* = 8.8 Hz, 2H, C*H_Ar_*), 6.65 (d, *J* = 8.2 Hz, 4H, C*H_Ar_*), 6.39–6.47 (m, 4H, C*H_Ar_*), 4.06–4.23 (m, 20H, C*H_2_*-X), 3.87–3.93 (m, 4H, C*H_2_*), 3.60 (br. s, 4H, N*H_2_*), 2.56–2.63 (m, 4H, C*H_2_*), 1.67–1.68 (m, 8H, C*H_2_*), 1.21 (t, *J* = 7.6 Hz, 12H, C*H_3_*) ppm; ^13^C NMR (CDCl_3_, 75 MHz) δ 171.4, 154.0, 144.6, 144.3, 140.6, 132.3, 129.2 115.2, 114.9, 107.0, 106.2, 68.3, 68.1, 60.8, 53.5, 34.8, 28.9, 28.1, 14.1 ppm; HRMS–FD (*m*/*z*): [M]^+^ calcd for C_50_H_66_N_4_O_12_, 914.4677; found, 914.4668.

**Azobenzene-BAPTA tetraester (1f)**. To a solution of **1e** (238 mg, 0.26 mmol) in 128 mL pyridine was added CuCl (260 mg, 2.62 mmol), the flask was left open to the air and the resulting mixture was stirred at room temperature for 48 h. Then, the solvent was removed in vacuo and column chromatography (SiO_2_, EtOAc/petroleum ether 2:8, v/v) yielded the cyclized azobenzene (37 mg, 16%). ^1^H NMR (CDCl_3_, 300 MHz) δ 7.88 (d, *J* = 8.0 Hz, 4H, C*H_Ar_*), 7.37 (d, *J* = 9.0 Hz, 4H, C*H_Ar_*), 6.80 (d, *J* = 8.7 Hz, 2H, C*H_Ar_*), 6.38–6.48 (m, 4H, C*H_Ar_*), 4.21 (s, 4H, C*H_2_*), 4.19 (s, 8H, C*H_2_*), 4.12 (q, *J* = 7.2 Hz, C*H_2_*), 3.87–3.97 (m, 4H, C*H_2_*), 2.74–2.83 (m, 4H, C*H_2_*), 1.87–1.92 (m, 8H, C*H_2_*), 1.21 (t, *J* = 7.2 Hz, 12H) ppm; ^13^C NMR (CDCl_3_, 75 MHz) δ 171.4, 154.0, 151.2, 145.6, 144.6, 140.6, 129.1, 122.8, 115.0, 107.1, 106.2, 68.1, 60.8, 53.5, 35.5, 29.7, 29.0, 27.8, 14.1 ppm; HRMS–FD (*m*/*z*): [M]^+^ calcd for C_50_H_62_N_4_O_12_, 910.4364; found, 910.4368.

**Hydrolyzed azobenzene-BAPTA (1).** To a solution of **1f** (87 mg, 0.040 mmol) in 11 mL THF/MeOH 5:1 (v/v), LiOH∙H_2_O (51 mg, 1.21 mmol) was added and the reaction mixture stirred overnight at room temperature. The solvent was removed in vacuo (at 30 °C) and the residue was sonicated briefly in 10 mL DCM/EtOAc 1:1 (v/v) and filtered. The solid is solubilized in Milli-Q water and acidified with HCl. The resulting suspension was centrifuged, the supernatant removed and the precipitate washed with a small volume of water. Then, the precipitate was redissolved by adding a small volume of a KOH solution and the solvent removed. ^1^H NMR (DMSO-*d*_6_, 300 MHz) δ 8.09 (d, *J* = 3 Hz, 2H, C*H_Ar_*), 7.82 (dd, *J* = 9 Hz and 3 Hz, 2H, C*H_Ar_*), 7.70 (d, *J* = 9 Hz, 4H, C*H_Ar_*), 7.40 (d, *J* = 9 Hz, 2H, C*H_Ar_*), 6.89 (d, *J* = 9.0 Hz, 4H, C*H_Ar_*), 4.52 (s, 4H, C*H_2_*), 4.27 (s, 8H, C*H_2_*), 3.63 (t, *J* = 7 Hz, 4H, C*H_2_*), 3.41 (t, *J* = 7 Hz, 4H, C*H_2_*), 1.75 (m, 4H, C*H_2_*), 1.51 (m, 4H, C*H_2_*) ppm.

## Supporting Information

File 1 Electronic absorption spectra, NMR and mass spectra.
