# Supplementary material for: Photoreversible stretching of a BAPTA chelator marshalling Ca2+-binding in aqueous media
Source: Beilstein J Org Chem. 2019 Nov 21;15:2801–11. doi: 10.3762/bjoc.15.273 (PMC6880838; doi:10.3762/bjoc.15.273)
Supplement: File 1 — Electronic absorption spectra, NMR and mass spectra. [file Beilstein_J_Org_Chem-15-2801-s001.pdf]

BEILSTEIN JOURNAL OF ORGANIC CHEMISTRY

Supporting Information

for

Photoreversible stretching of a BAPTA chelator marshalling Ca^{2+}-binding in aqueous media

Aurélien Ducrot, Arnaud Tron, Robin Bofinger, Ingrid Sanz Beguer, Jean-Luc Pozzo and Nathan D. McClenaghan

Beilstein J. Org. Chem. **2019,** *15,* 2801–2811. doi:10.3762/bjoc.15.273

Electronic absorption spectra, NMR and mass spectra

License and Terms: This is a supporting information file under the terms of the Creative Commons Attribution License (http://creativecommons.org/licenses/by/4.0). Please note that the reuse, redistribution and reproduction in particular requires that the authors and source are credited.
The license is subject to the *Beilstein Journal of Organic Chemistry* terms and conditions: (https://www.beilstein-journals.org/bjoc)

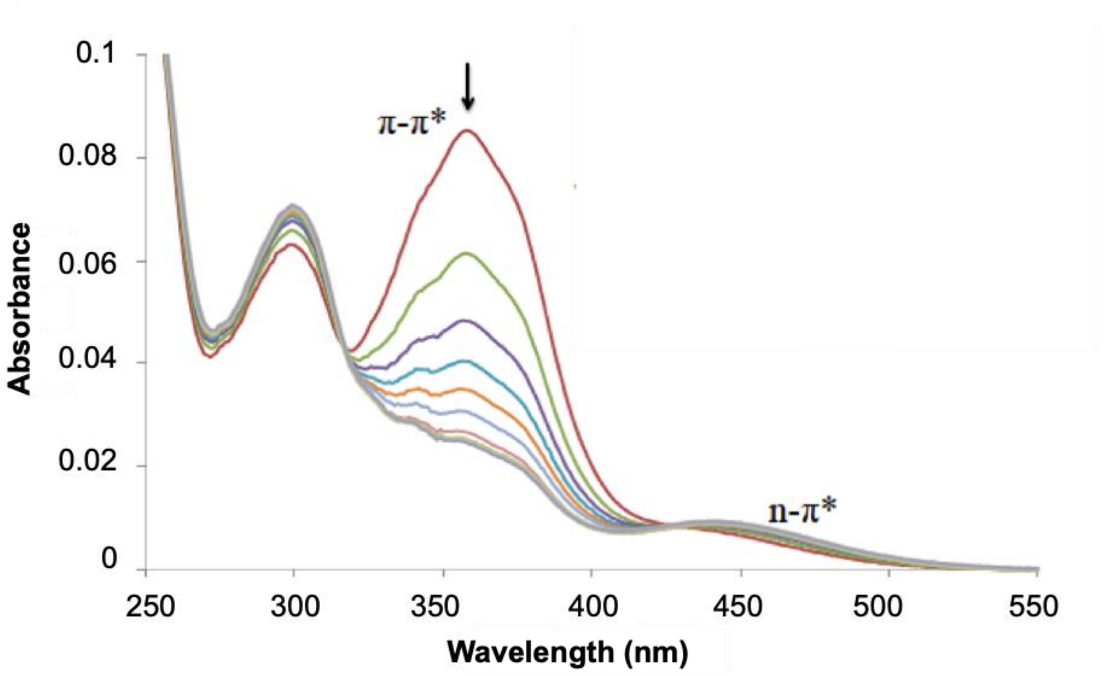

Figure S1: Electronic absorption spectra of **1f*E*** at a concentration of 40 μM in CH_3CN. Upon irradiation at 365 nm, the decrease of the π–π* band and the increase of the *n*–π* band via changes at 362 nm and 460 nm, respectively, were observed.

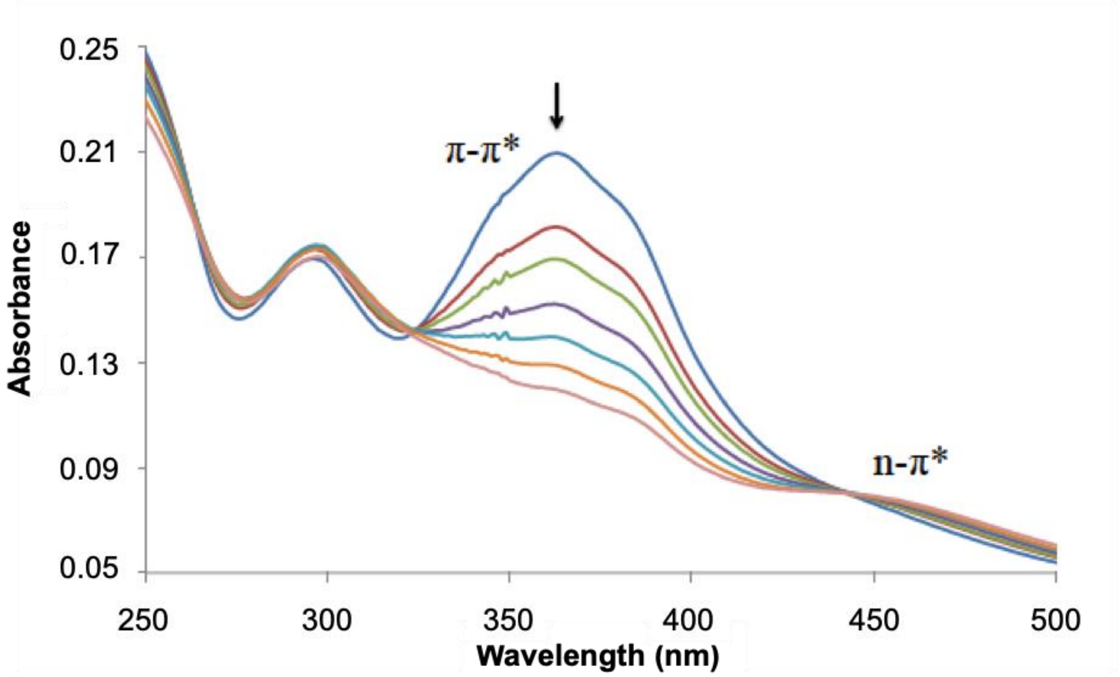

Figure S2: Electronic absorption spectra of **1*E*** at a concentration of 40 μM in 0.03 M MOPS buffer at pH 7.2 in the presence of KCl (0.1 M), EGTA (0.011 M) and Ca^{2+} (4.4 mM). Upon irradiation at 365 nm, the decrease of the π–π* band and the increase of the *n*–π* band via changes at 362 nm and 460 nm, respectively, were observed.

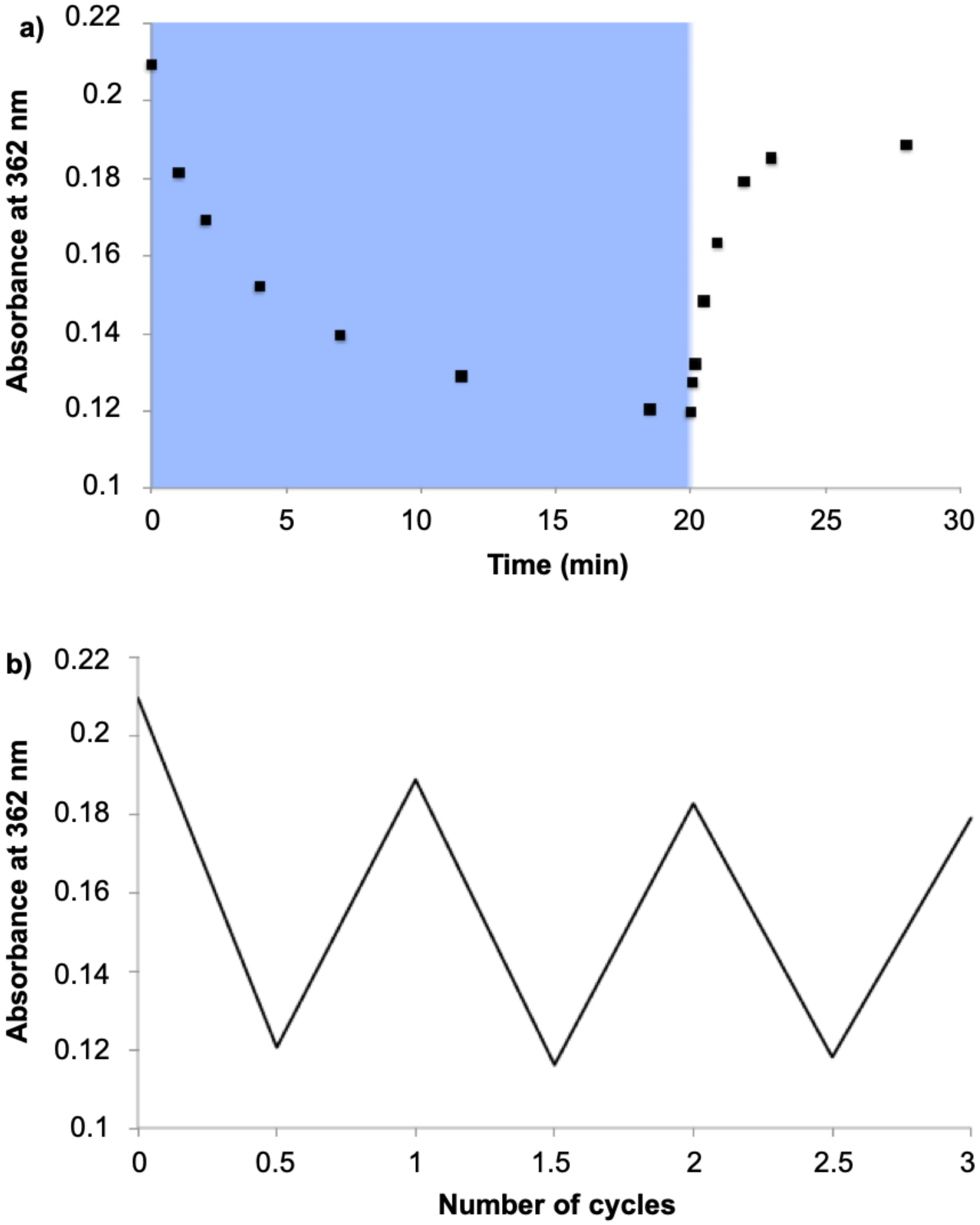

Figure S3: a) Multiple *trans*/*cis*-cycles of **1*E*** (40 μM) indicated by absorption changes at 362 nm, in aqueous 0.03 M MOPS buffer at pH 7.2 in the presence of KCl (0.1 M), EGTA (0.011 M) and Ca^{2+} (4.4 mM). Each cycle corresponds to irradiation at 365 nm (blue), followed by thermal return at room temperature (white). b) Fatigue study of **1*E*** in the presence of calcium after 3 cycles.

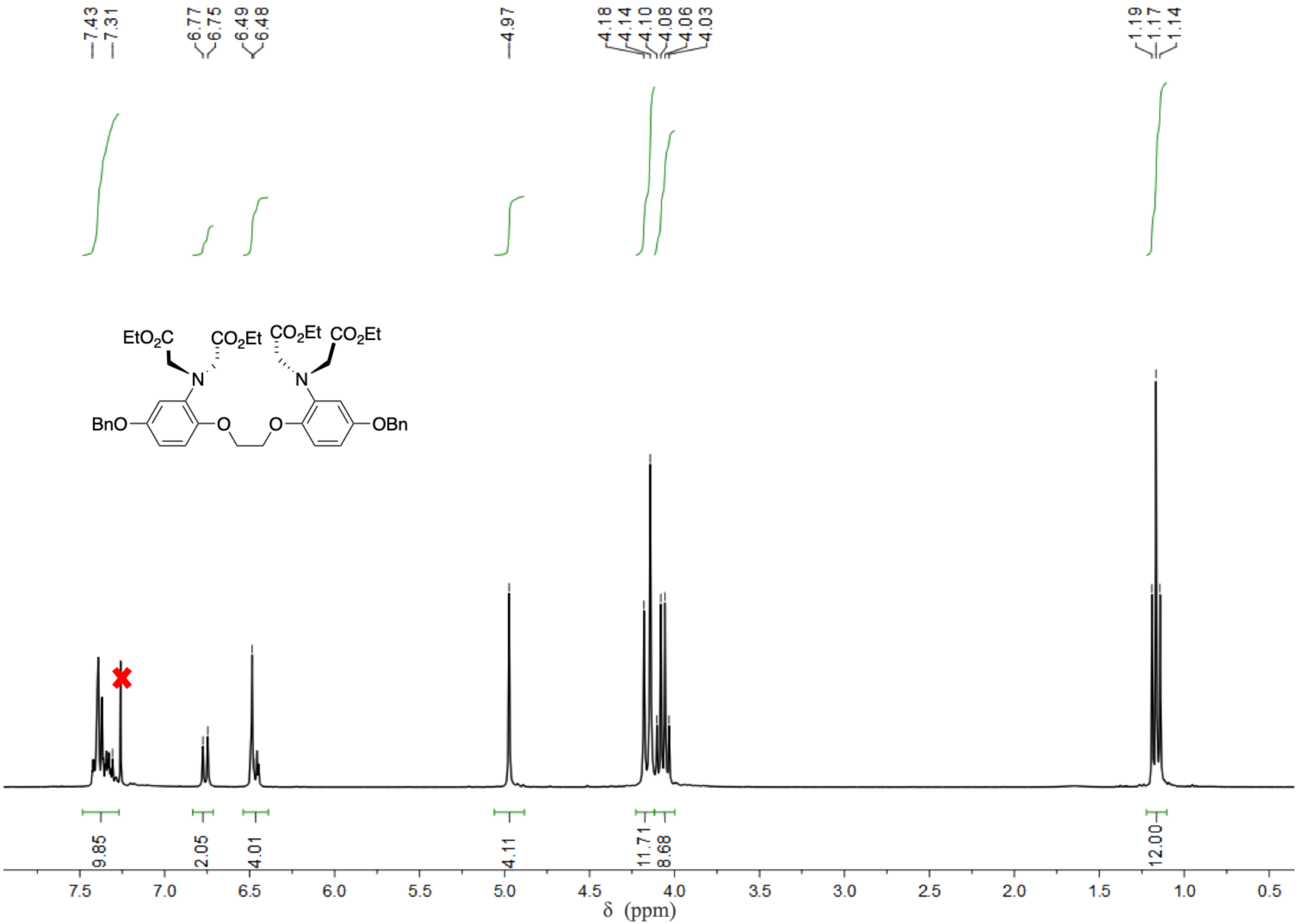

Figure S4: ^{1}H NMR spectrum of the **1b** in $CDCl_3$ at 298 K on a 300 MHz spectrometer (red X corresponds to chloroform).

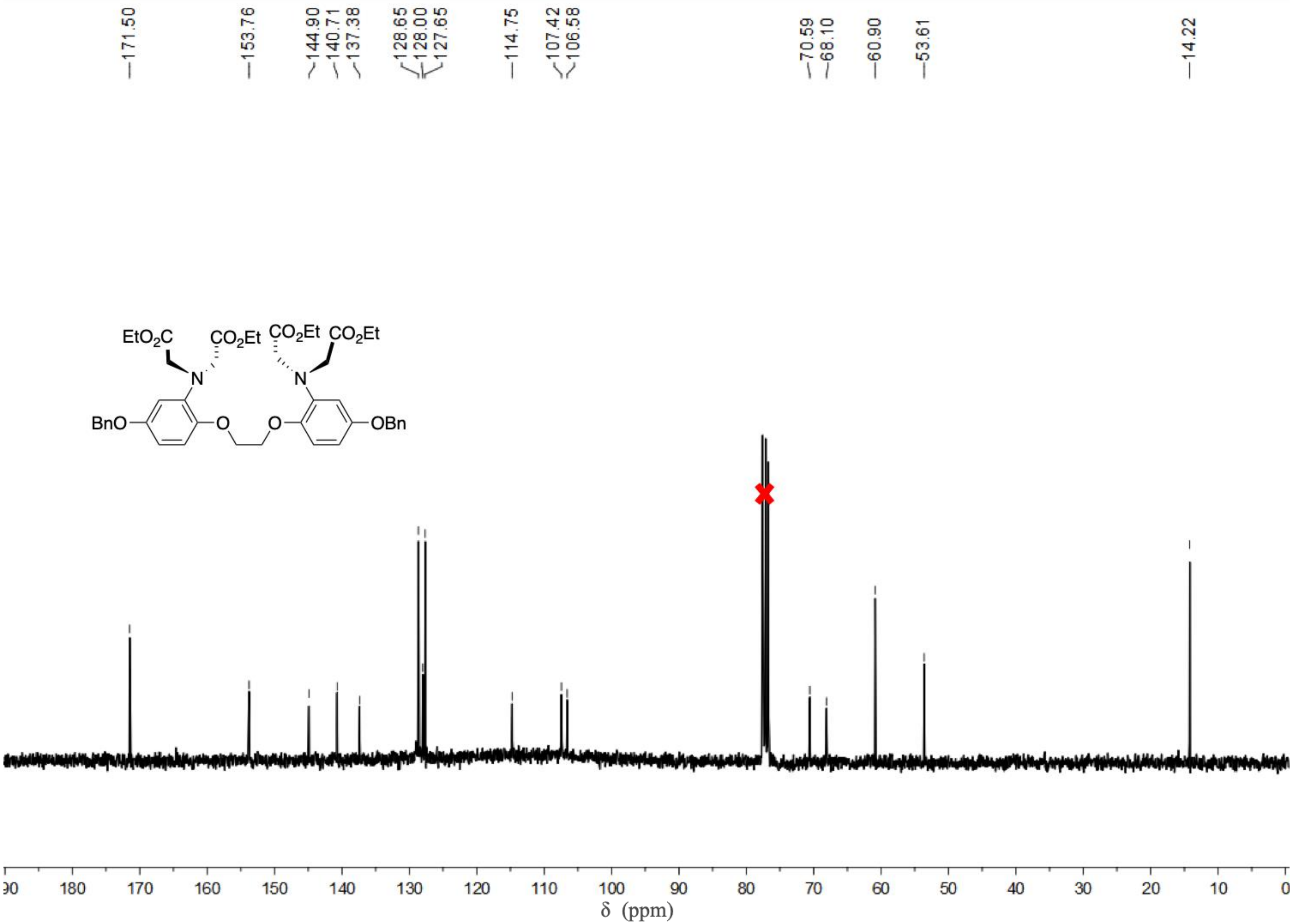

Figure S5: ^{13}C NMR spectrum of the **1b** in $CDCl_3$ at 298 K on a 300 MHz spectrometer (red X corresponds to chloroform).

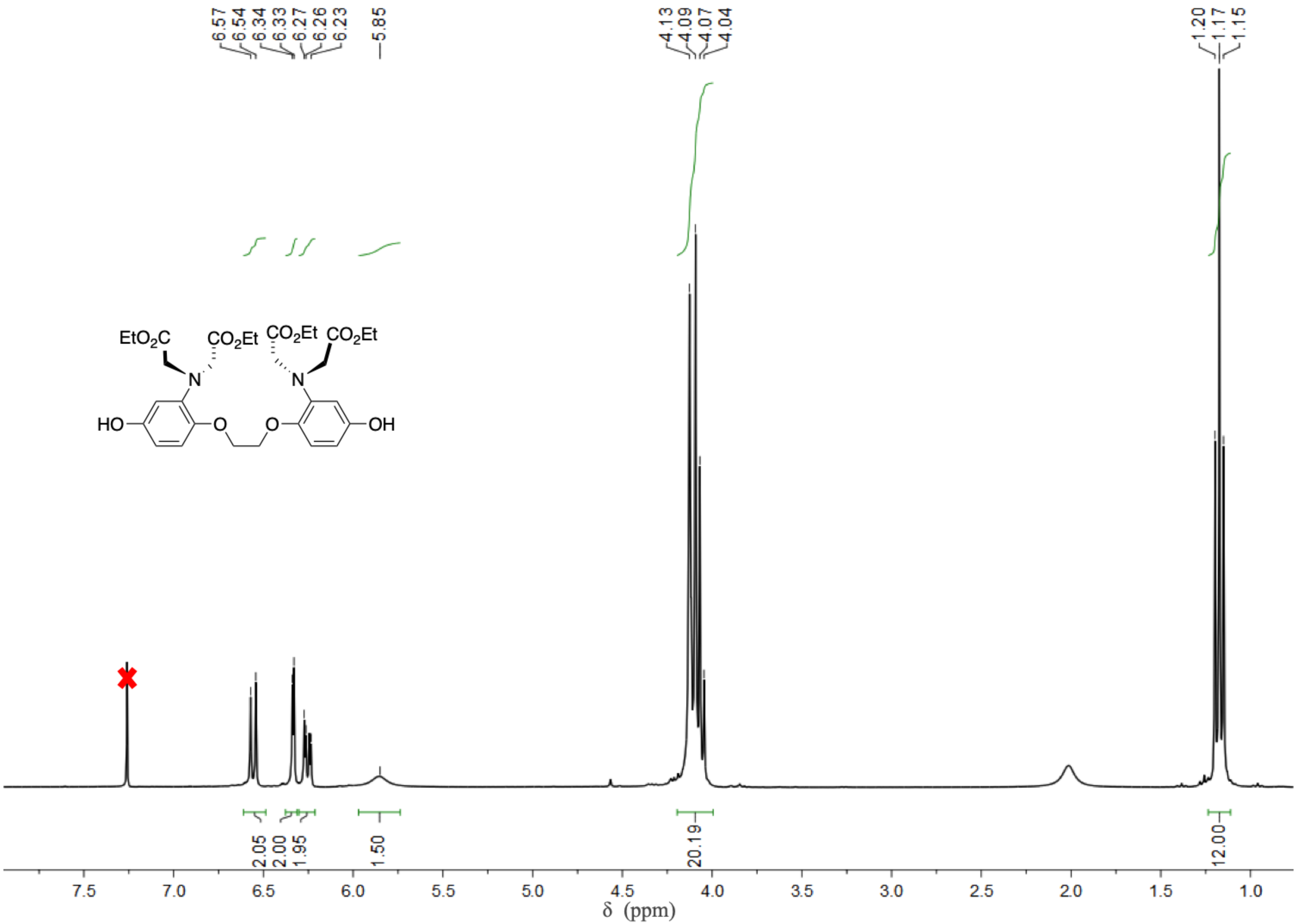

Figure S6: ^{1}H NMR spectrum of the **1c** in $CDCl_3$ at 298 K on a 300 MHz spectrometer (red X corresponds to chloroform).

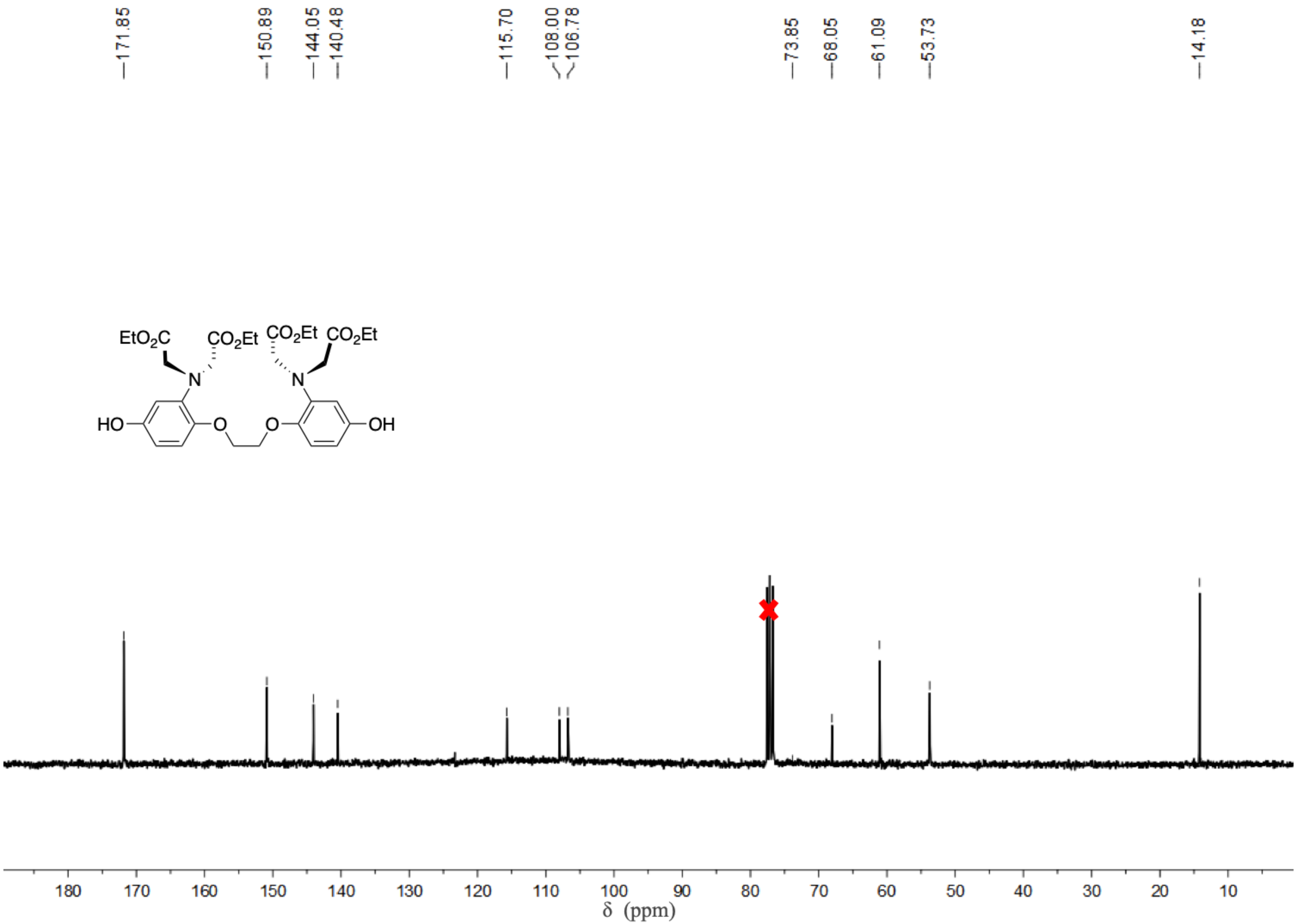

Figure S7: ^{13}C NMR spectrum of the **1c** in $CDCl_3$ at 298 K on a 300 MHz spectrometer (red X corresponds to chloroform).

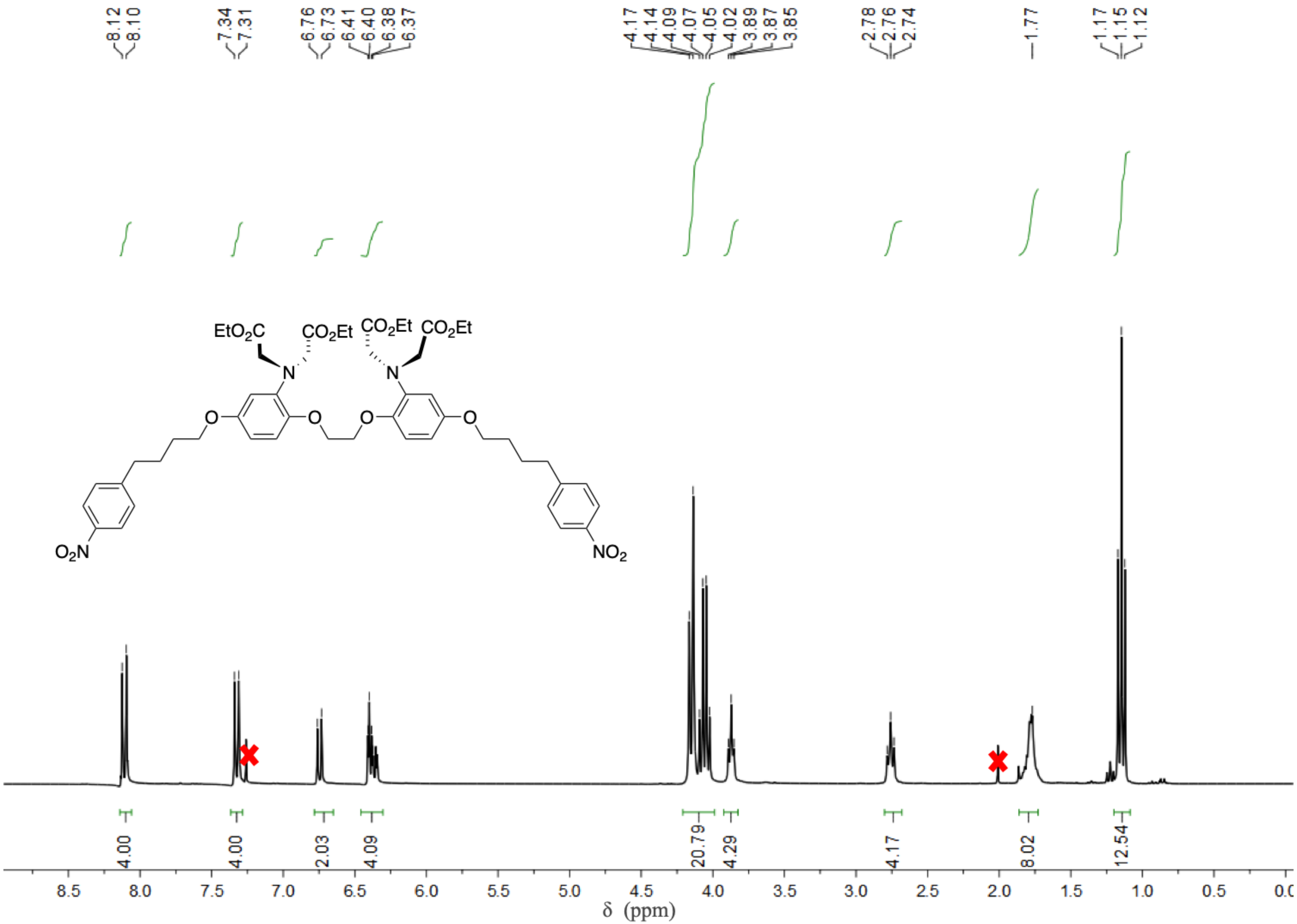

Figure S8: ^{1}H NMR spectrum of the **1d** in $CDCl_3$ at 298 K on a 300 MHz spectrometer (red X corresponds to chloroform and acetone).

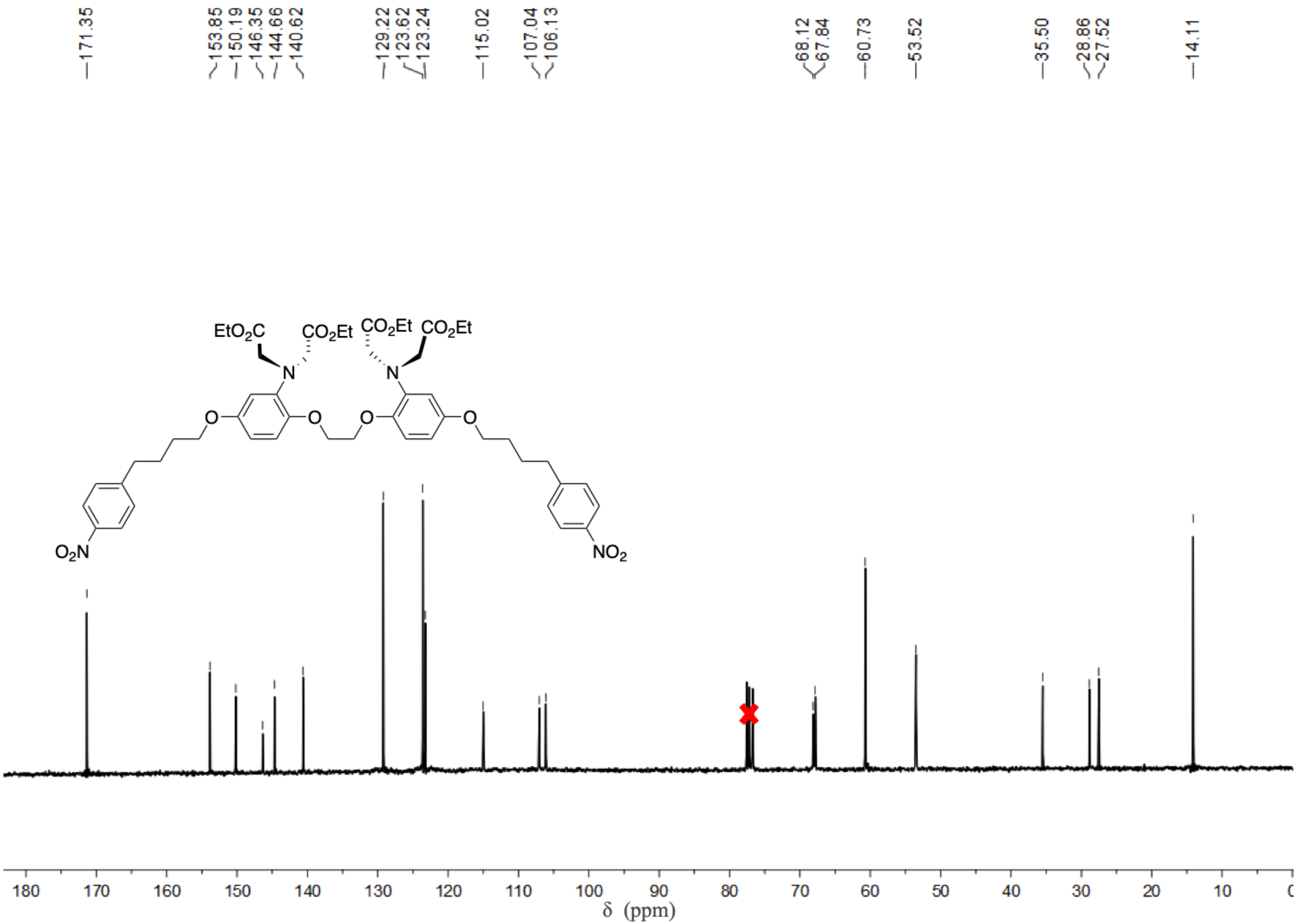

Figure S9: ^{13}C NMR spectrum of the **1d** in $CDCl_3$ at 298 K on a 300 MHz spectrometer (red X corresponds to chloroform).

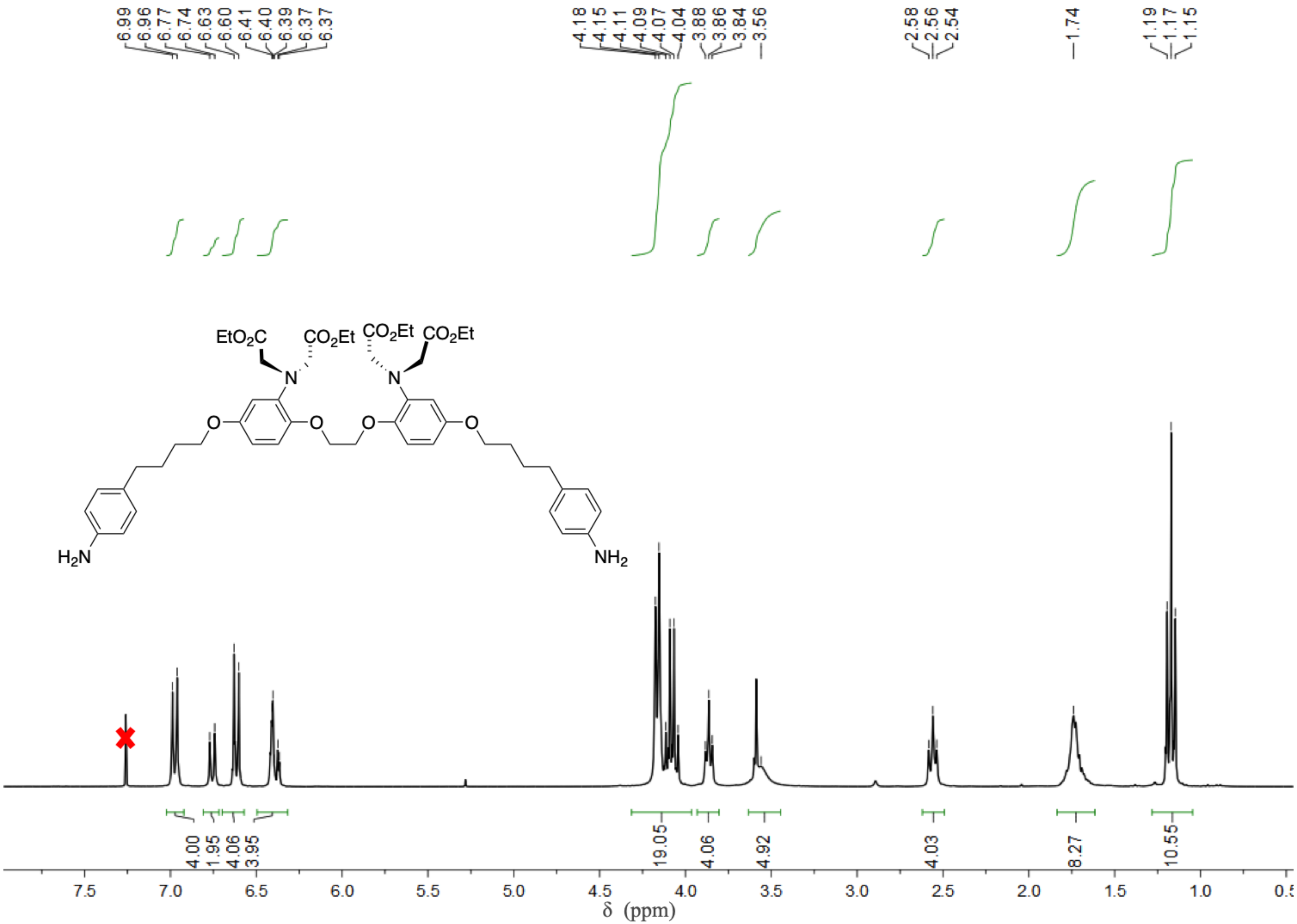

Figure S10: ^{1}H NMR spectrum of the **1e** in $CDCl_3$ at 298 K on a 300 MHz spectrometer (red X corresponds to chloroform).

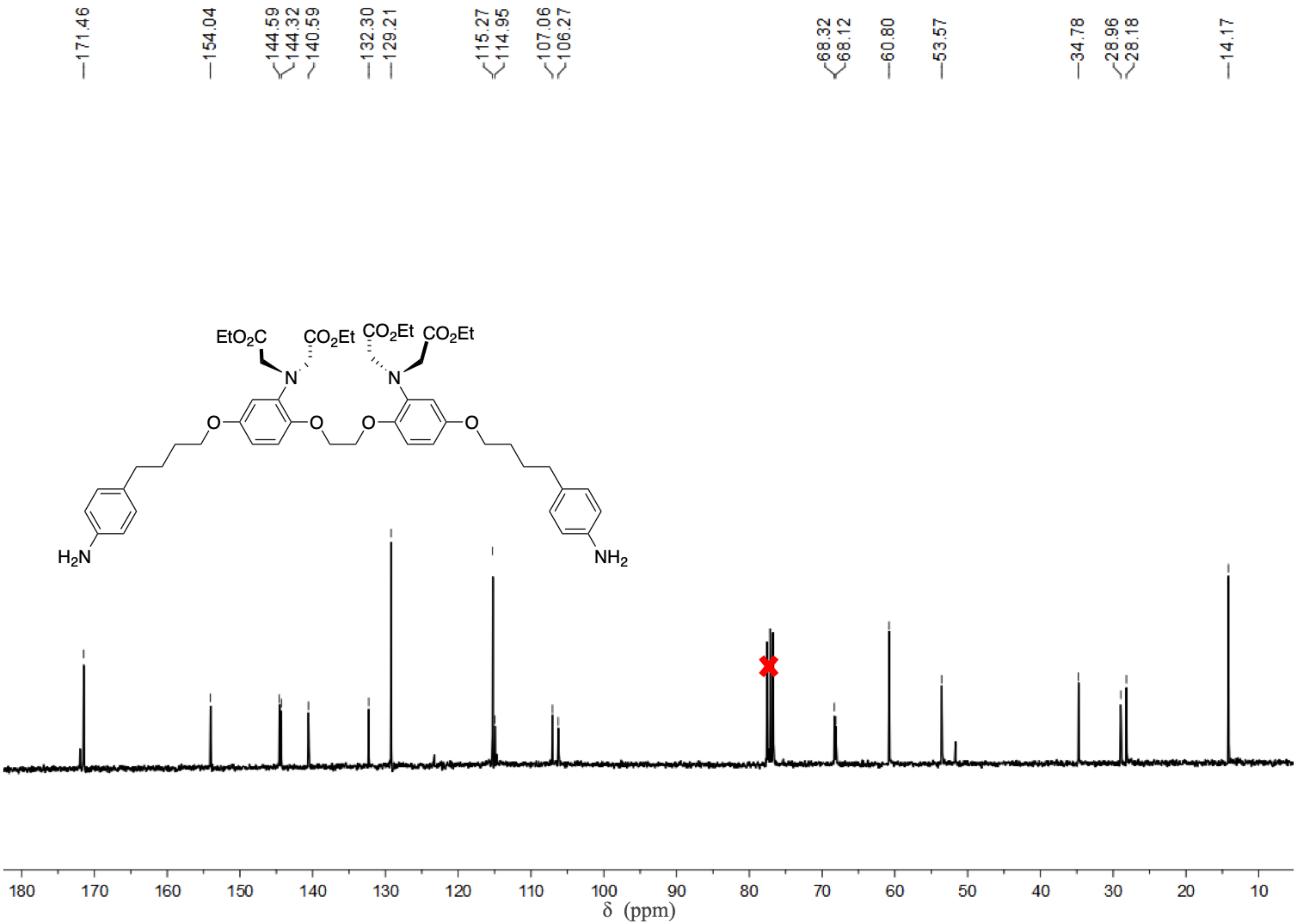

Figure S11: ^{13}C NMR spectrum of the **1e** in $CDCl_3$ at 298 K on a 300 MHz spectrometer (red X corresponds to chloroform).

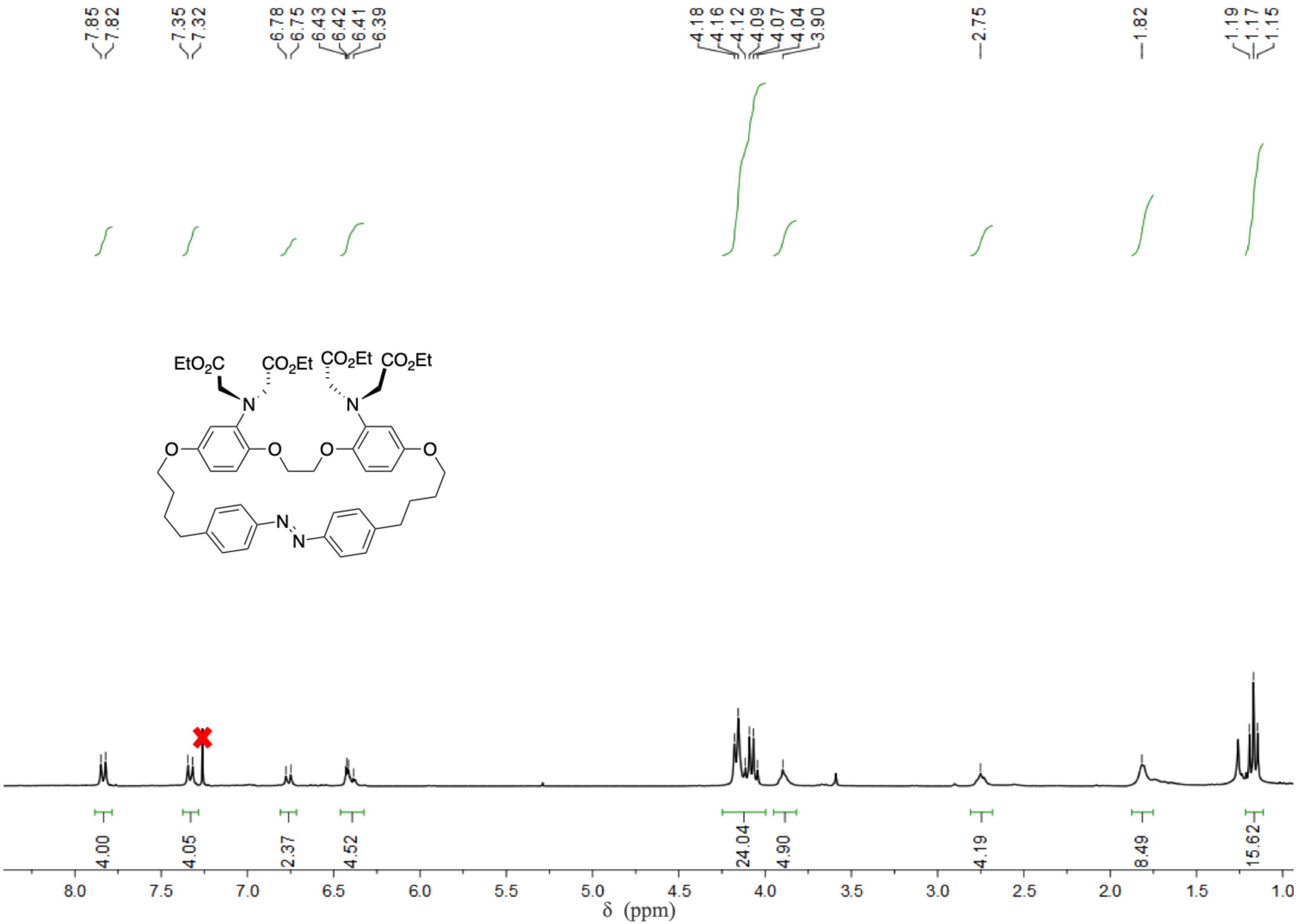

Figure S12: ^{1}H NMR spectrum of the **1f** in $CDCl_3$ at 298 K on a 300 MHz spectrometer (red X corresponds to chloroform).

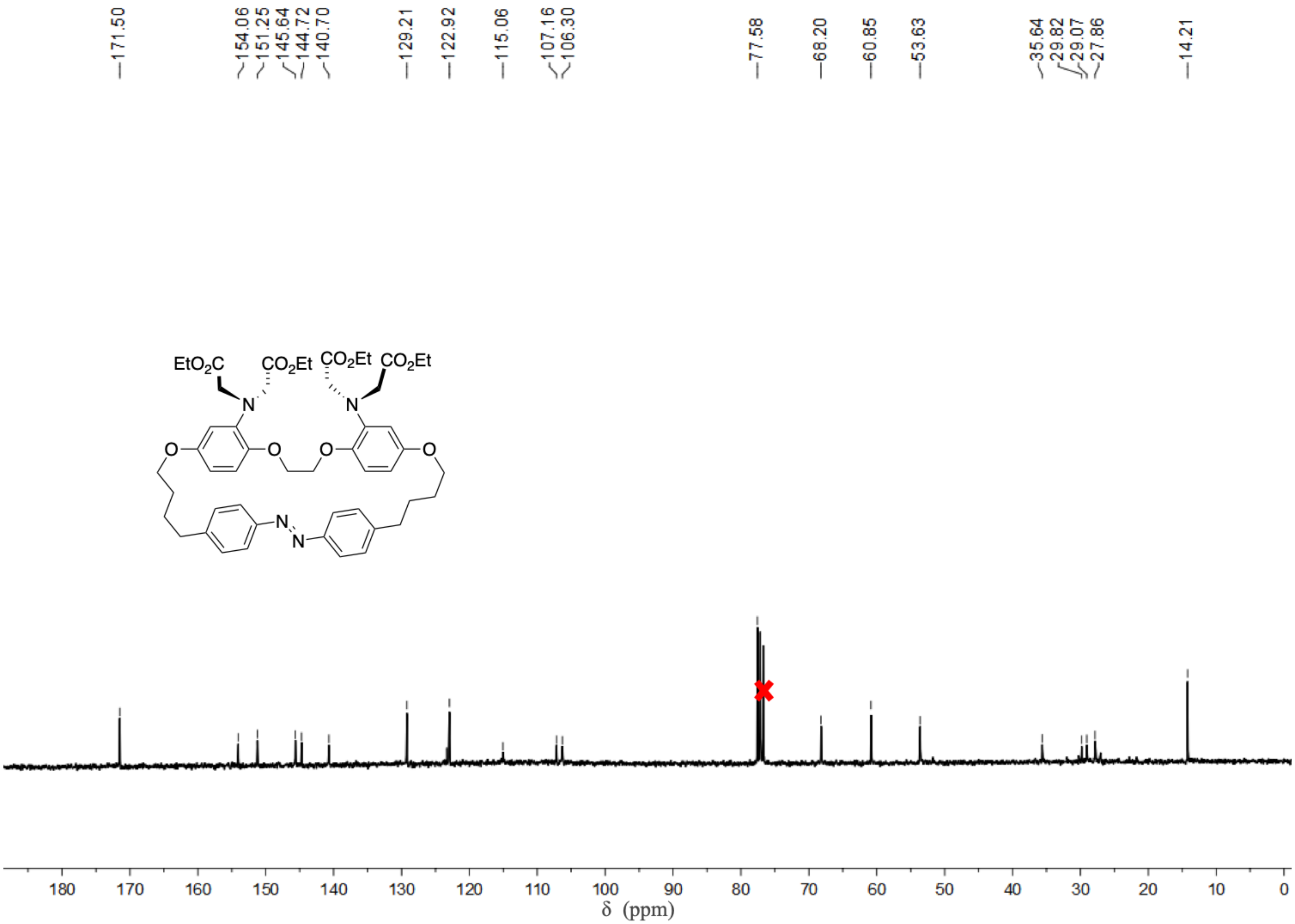

Figure S13: ^{13}C NMR spectrum of the **1f** in $CDCl_3$ at 298 K on a 300 MHz spectrometer (red X corresponds to chloroform).

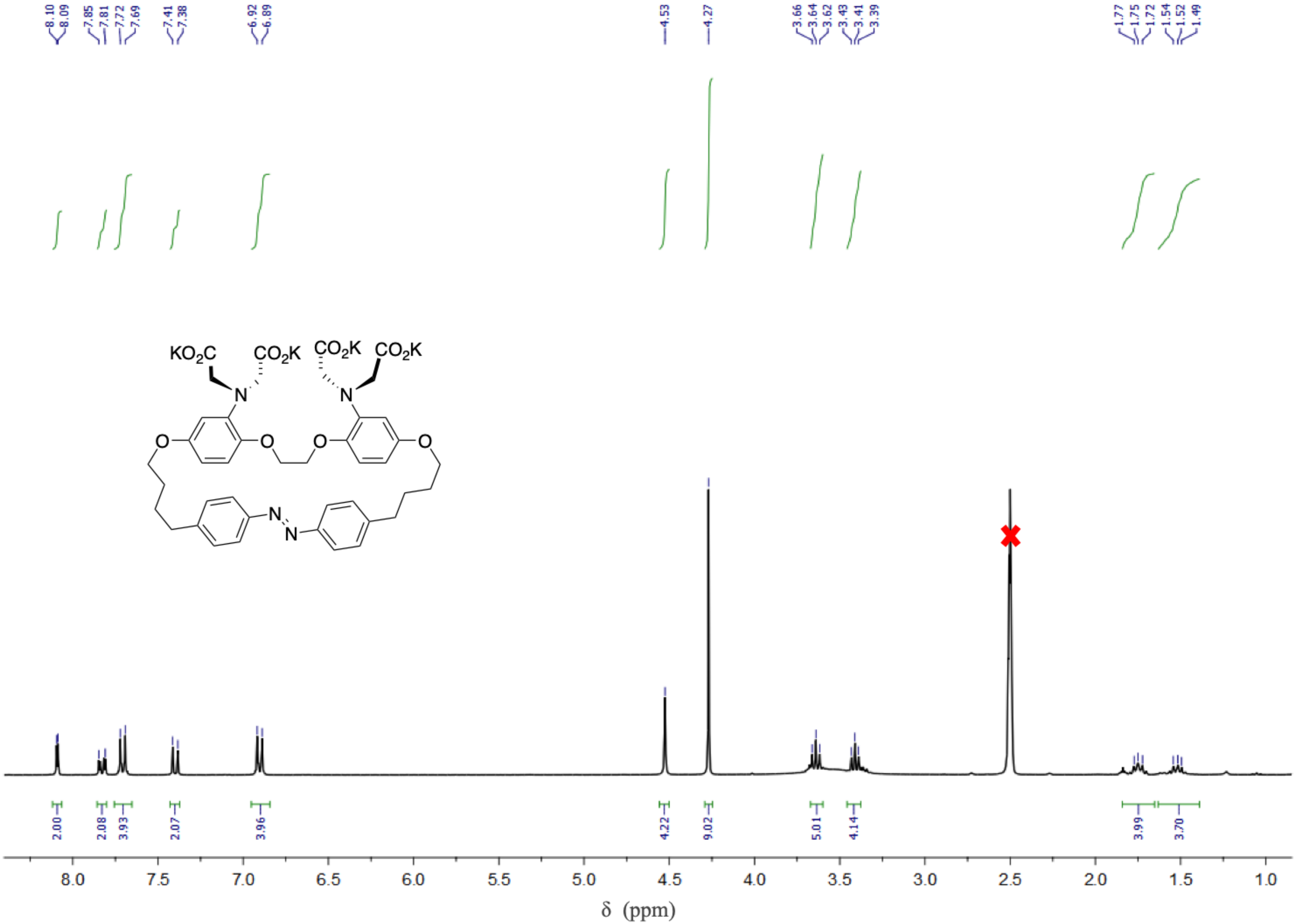

Figure S14: ^{1}H NMR spectrum of the **1** in DMSO-d_6 at 298 K on a 300 MHz spectrometer (red X corresponds to DMSO).
